# Use of basal insulin in the management of adults with type 2 diabetes: An Asia‐Pacific evidence‐based clinical practice guideline

**DOI:** 10.1111/1753-0407.13392

**Published:** 2023-04-23

**Authors:** Linong Ji, Yingying Luo, Yong Mong Bee, Jun Xia, Khue Thy Nguyen, Weigang Zhao, Liming Chen, Siew Pheng Chan, Chaicharn Deerochanawong, Soo Lim, Daisuke Yabe, Margaret McGill, Ketut Suastika, Xiaoying Li, Alice Pik Shan Kong, Wei Chen, Zhan Zhao, Chenchen Xu, Marisa Deodat, Xiaomei Yao

**Affiliations:** ^1^ Department of Endocrinology and Metabolism Peking University People's Hospital Beijing China; ^2^ Department of Endocrinology Singapore General Hospital Singapore Singapore; ^3^ Nottingham Ningbo GRADE Centre University of Nottingham Ningbo China Zhejiang China; ^4^ Academic Unit of Lifespan and Population Health, School of Medicine The University of Nottingham Nottingham UK; ^5^ Ho Chi Minh University of Medicine and Pharmacy Medic Medical Center Ho Chi Minh City Vietnam; ^6^ Department of Endocrinology Peking Union Medical College Hospital Beijing China; ^7^ Chu Hsien‐I Memorial (Metabolic Diseases) Hospital & Tianjin Institute of Endocrinology Tianjin Medical University Tianjin China; ^8^ Department of Medicine, Faculty of Medicine University of Malaya Kuala Lumpur Malaysia; ^9^ College of Medicine Rangsit University Bangkok Thailand; ^10^ Department of Internal Medicine Seoul National University College of Medicine and Seoul National University Bundang Hospital Seongnam South Korea; ^11^ Departments of Diabetes, Endocrinology and Metabolism/Rheumatology and Clinical Immunology Gifu University Graduate School of Medicine Gifu Japan; ^12^ Center for One Medicine Innovative Translational Research Gifu University Institute for Advanced Study Gifu Japan; ^13^ Diabetes Centre, Royal Prince Alfred Hospital Faculty of Medicine and Health University of Sydney Sydney Australia; ^14^ Division of Endocrinology and Metabolism, Department of Internal Medicine, Faculty of Medicine Udayana University, Prof. IGNG Ngoerah Hospital Denpasar Indonesia; ^15^ Department of Endocrinology and Metabolism, Zhongshan Hospital Fudan University Shanghai China; ^16^ Division of Endocrinology, Department of Medicine and Therapeutics The Chinese University of Hong Kong, Hong Kong Special Administrative Region Hong Kong China; ^17^ Department of Clinical Nutrition, Department of Health Medicine Chinese Academy of Medical Sciences‐Peking Union Medical College, Peking Union Medical College Hospital Beijing China; ^18^ Tianjin Tiantian Biotechnology Co., Ltd Tianjin China; ^19^ Michael G. DeGroote Cochrane Canada and McMaster GRADE Centres McMaster University Hamilton Ontario Canada; ^20^ Department of Health Research Methods, Evidence, and Impact McMaster University Hamilton Ontario Canada; ^21^ Center for Clinical Practice Guideline Conduction and Evaluation Children's Hospital of Fudan University Shanghai China

**Keywords:** Asia‐Pacific region, basal insulin, clinical thresholds, evidence‐based clinical practice guideline, systematic review, type 2 diabetes, 亚太地区, 基础胰岛素, 临床阈值, 循证临床实践指南, 系统回顾, 2型糖尿病

## Abstract

The objective of this study was to provide recommendations regarding effectiveness, safety, optimal starting dose, optimal maintenance dose range, and target fasting plasma glucose of five basal insulins (glargine U‐300, degludec U‐100, glargine U‐100, detemir, and insulin protamine Hagedorn) in insulin‐naïve adult patients with type 2 diabetes in the Asia‐Pacific region. Based on evidence from a systematic review, we developed an Asia‐Pacific clinical practice guideline through comprehensive internal review and external review processes. We set up and used clinical thresholds of trivial, small, moderate, and large effects for different critical and important outcomes in the overall certainty of evidence assessment and balancing the magnitude of intervention effects when making recommendations, following GRADE methods (Grading of Recommendations, Assessment, Development, and Evaluation). The AGREE (Appraisal of Guidelines, Research and Evaluation) and RIGHT (Reporting Items for practice Guidelines in HealThcare) guideline reporting checklists were complied with. After the second‐round vote by the working group members, all the recommendations and qualifying statements reached over 75% agreement rates. Among 44 contacted external reviewers, we received 33 clinicians' and one patient's comments. The overall response rate was 77%. To solve the four research questions, we made two strong recommendations, six conditional recommendations, and two qualifying statements. Although the intended users of this guideline focused on clinicians in the Asia‐Pacific region, the eligible evidence was based on recent English publications. We believe that the recommendations and the clinical thresholds set up in the guideline can be references for clinicians who take care of patients with type 2 diabetes worldwide.

## Graphical abstract

**Highlights**

- This is a systematic review‐based clinical practice guideline. After internal and external review, two strong recommendations, six conditional recommendations, and one qualifying statement were made for insulin‐naive adult patients with type 2 diabetes. Although the guideline authors came from the Asia‐Pacific region, the eligible evidence was based on recent English publications. The recommendations and the clinical thresholds set up in the guideline can be used as references for clinicians worldwide.

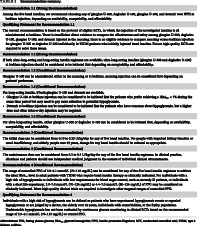

## 摘要

**【提要】** 为亚太地区初次使用胰岛素的成人2型糖尿病患者提供关于5种基础胰岛素(甘精胰岛素U‐300、德谷胰岛素U‐100、甘精胰岛素U‐100型、地特胰岛素和中性鱼精蛋白胰岛素)的有效性、安全性、最佳起始剂量、最佳维持剂量范围和目标空腹血浆葡萄糖的推荐。基于系统回顾的证据，本综述通过全面的内部审核和外部审核流程制定了亚太地区临床实践指南。本综述在整体证据确定性评估中为不同重要和关键结局设置了微小、小、中等和大效应的临床阈值，并遵循GRADE(评估、发展、评价、建议的分级)方法来平衡干预效果的大小以进行推荐。本综述遵守了AGREE(指南评估、研究和评估)和RIGHT(卫生保健实践指南报告项目)指南报告检查清单。在工作组成员进行第二轮投票后，所有推荐和限定性陈述的一致率均达到了75%以上。在联系的44名外部评审人员中，本研究收到了33名临床医生和1名患者的意见。总体应答率为77%。为解决4个研究问题，本研究提出了2个强烈推荐、6个有条件推荐和2个限定性陈述。尽管本指南的预期用户主要是亚太地区的临床医生，但所选的证据基于最近的英文出版物。本综述提示，指南中制定的推荐和临床阈值可以成为全球关注2型糖尿病患者的临床医生的参考。

## 1 INTRODUCTION

Achieving target glycemic control without causing hypoglycemia is crucial for patients with type 2 diabetes mellitus (T2DM), as it is linked to preventing complications and improving their long‐term quality of life. Many T2DM patients eventually require injectable glucose‐lowering therapy, such as glucagon‐like peptide‐1 receptor agonist or insulin when a combination of rally administered antihyperglycemic agents (OHAs) fails to maintain the desired glycemic targets. Among the various insulin options, basal insulin is recommended at the initiation of insulin therapy.^1^ To date, there are five basal insulin preparations available for use in patients with diabetes mellitus in the Asia‐Pacific region: (1) ultra‐long‐acting insulin: glargine U‐300, degludec U‐100; (2) long‐acting insulin: glargine U‐100, detemir; and (3) intermediate‐acting insulin: insulin protamine Hagedorn (NPH). After a quick literature search for English publications, there are no systematic review‐based clinical practice guidelines in the Asia‐Pacific region on the use of basal insulin in insulin‐naive adults with T2DM who are poorly controlled on OHAs. For example, there is a Delphi‐based consensus guideline on basal insulins in Asia.^1^ The American Diabetes Association published “Standards of Medical Care in Diabetes—2021,” which is not a systematic review‐based guideline.^2^ Thus, 16 clinical experts from Australia, China, Indonesia, Japan, Malaysia, Singapore, South Korea, Thailand, and Vietnam worked with three methodologists and a research team to develop this guideline focusing on answering the following four research questions. Degludec U‐200 was produced for patients with T2DM who need a very high insulin dose and is unavailable in most countries. Therefore, it is not included in this guideline.

Q1. What are the differences in the effectiveness and safety among the five basal insulin preparations after the initiation of insulin therapy in the target population?

Q2. What are the optimal starting doses (U/kg/day) and time of administration (morning versus bedtime) of five basal insulins?

Q3. Among the target population, what is the optimal maintenance dose (U/kg/day) that achieves target fasting plasma glucose (FPG)?

Q4. After initiation of any of the five basal insulins, what range of the target FPG can lead to the ideal glycated hemoglobin (HbA_1c_) level (ie, <7.0%) in the target population?

### 1.1 Target population

Nonpregnant adults with T2DM who are unable to achieve target glycemic goals (HbA_1c_ > 7%) on OHAs.

### 1.2 Intended users

Endocrinologists, general internal medicine physicians, family doctors, diabetes specialist nurses, and other clinicians who are involved in the care of the target patients in the Asia‐Pacific region.

## 2 METHODS

The process of developing this evidence‐based clinical practice guideline includes a systematic review, interpretation of the evidence by the core group (two clinical experts [L.J., Y.L.] and one methodologist [X.Y.]), drafting recommendations, an internal review by the working group, and an external review by external reviewers who were nominated by the working group members. The GRADE methodology (Grading of Recommendations, Assessment, Development, and Evaluation) was used to assess the certainty of evidence and make recommendations.^3^ Two guideline reporting checklists have been adhered to: AGREE (Appraisal of Guidelines, Research and Evaluation) and RIGHT (Reporting Items for practice Guidelines in HealThcare).^4^, ^5^, ^6^ The systematic review manuscript, including the literature search, study review process, etc., is submitted separately.^7^ This guideline has been registered on the Guideline International Network website.^8^

### 2.1 Ranking importance of outcomes

Glycemic control as measured by HbA_1c_ and incidence of any, nocturnal, and severe hypoglycemia were rated as “critical” outcomes; time in range, FPG control, cost‐effectiveness, weight gain, and patient‐reported outcomes were rated as “important” outcomes. When making recommendations, we placed more emphasis on the effects and certainty of evidence of the critical outcomes.

### 2.2 Clinical thresholds for critical and important outcomes

Based on the GRADE guidance,^9^ during the medical literature search, we found two publications that mentioned minimal important difference threshold for the change of HbA_1c_ (%), which was stated as 0.5%.^10^, ^11^ However, we did not find other clinical thresholds of trivial, small, moderate, and large effects for any critical outcomes and important outcomes, which are the base to assess the imprecision domain of overall certainty of evidence and to balance the magnitude of intervention effects when making recommendations. Thus, the core group set up the clinical thresholds for the “critical” and “important” outcomes (Table 1). These clinical thresholds were circulated among the working group to vote on to obtain a consensus (>85% agreement rate for each outcome) (Appendix Table S1).

**TABLE 1:** Clinical thresholds for critical and important outcomes.

Outcomes	Trivial effect	Small effect	Moderate effect	Large effect
HbA_1c_ < 7%	<30 per 1000	30–50 per 1000	>50–100 per 1000	>100 per 1000
Mean HbA_1c_ change value (from baseline to the end of the study), %	<0.4	0.4–1.0	>1.0–1.5	>1.5
Hypoglycemia <3.0 mmol/L	<20 per 1000	20–50 per 1000	>50–80 per 1000	>80 per 1000
Hypoglycemia <3.9 mmol/L	<50 per 1000	50–100 per 1000	>100–150 per 1000	>150 per 1000
Nocturnal hypoglycemia, hypoglycemia <3.0 or 3.9 mmol/L	<20 per 1000	20–50 per 1000	>50–80 per 1000	>80 per 1000
Severe hypoglycemia, hypoglycemia <3.0 or 3.9 mmol/L	<5 per 1000	5–10 per 1000	>10–20 per 1000	>20 per 1000
Mean FPG change value (from baseline to the end of the study)	<0.5 mmol/L	0.5–1 mmol/L	>1–1.5 mmol/L	>1.5 mmol/L
Mean weight change (from baseline to the end of the study)	<2%	2%–3%	>3%–5%	>5%

Abbreviations: FPG, fasting plasma glucose; HbA_1c_, glycated hemoglobin.

### 2.3 Internal review

The core group drafted the recommendations. The clinical experts in the working group reviewed, commented, and voted on the recommendations independently. The core group then revised the recommendations based on the working group's opinion. The revised recommendations were voted by the working group again until each recommendation reached an agreement of more than 75%.

### 2.4 External review

Each of the clinical experts from the core and working groups selected three potential external reviewers who can read English and are either intended users of basal insulin or patients with T2DM using basal insulin. The drafted clinical practice guideline and two brief online questionnaires (one for clinicians and one for patients) were sent to the external reviewers. We used the questionnaire templates for external reviewers from the Program in Evidence‐based Care program of McMaster University in Canada.^12^

## 3 RESULTS

### 3.1 Development of the first recommendations

The core group held five online meetings (2–5 h/meeting) to discuss the evidence from the systematic review, answer 12 methodological questions (such as balancing benefits and harms) for each recommendation based on the GRADE approach, and draft the first recommendation document.^3^ The main evidence and justification are reported here.

### 3.2 Internal review process

The first online survey with eight recommendations and eight qualifying statements was sent out to the clinical experts in the working group to vote on 13 September 2022 (Appendix Table S2). By 28 September, 14 of 16 clinical experts voted. Among the voting results, only five recommendations and qualifying statements reached an over 75% agreement rate. After the core group members' discussion and revision based on the working group members' comments, the second online survey with five recommendations and one qualifying statement for Q1, one qualifying statement for Q2, one qualifying statement for Q3, and one recommendation for Q4 was sent to the working group to vote again on 10 October (Appendix Table S3). By 21 October, all 16 working group clinical members voted, and all the recommendations and qualifying statements reached over 75% agreement rates. After discussion, the qualifying statement for Recommendation 1 was removed and two minor revisions were made before external review.

### 3.3 External review process

Two online questionnaires were sent out to 44 potential external reviewers on 11 November 2022. By 27 November, we received 33 clinicians' and one patient's review comments from nine countries. The overall response rate was 77%. The patient reviewer found that the recommendations were clear and unambiguous, and the recommendations considered issues and/or addressed outcomes that were important to patients (Appendix Table S4). The results of the questionnaire from clinicians are summarized in Appendix Table S5. The main comments regarding disagreement, concerns, and suggestions from external reviewers and the guideline authors' responses are summarized in Appendix Table S6. The final recommendations are listed in Table 2, which reflects the integration of the feedback obtained from the external reviewers, with approval by the guideline authors.

**TABLE 2:** Recommendations summary.

**Recommendation 1.1 (Strong Recommendation)**
Among the five basal insulins, we recommend choosing any of glargine U‐300, degludec U‐100, glargine U‐100, and detemir over NPH at bedtime injection, depending on availability, acceptability, and affordability.
**Qualifying Statement for Recommendation 1.1**
The current recommendation is based on the protocol of eligible RCTs, in which the injection of the investigated insulins is all administrated at bedtime. There is insufficient direct evidence to compare the effectiveness and safety among glargine U‐300, degludec U‐100, glargine U‐100, and detemir injected in the morning; there is no eligible evidence to compare morning versus bedtime injection for glargine U‐300 or degludec U‐100 individually in T2DM patients who initially injected basal insulins. Future high‐quality RCTs are required to solve these issues.
**Recommendation 1.2 (Strong Recommendation)**
If both ultra‐long‐acting and long‐acting insulin regimens are available, ultra‐long‐acting insulins (glargine U‐300 and degludec U‐100) at bedtime injection should be considered to be initiated first depending on acceptability and affordability.
**Recommendation 1.3 (Conditional Recommendation)**
Glargine U‐100 can be administered either in the morning or at bedtime. morning injection can be considered first depending on patients' preference.
**Recommendation 1.4 (Conditional Recommendation)**
For long‐acting insulin, if both glargine U‐100 and detemir are available, Glargine U‐100 at bedtime injection can be considered to be initiated first for patients who prefer achieving a HbA_1c_ < 7% during the same time period but may need to pay more attention to potential hypoglycemia. Detemir at bedtime injection can be considered to be initiated first for patients who have concerns about hypoglycemia, but a higher dose and often twice‐a‐day injection may be required.
**Recommendation 1.5 (Conditional Recommendation)**
For ultra‐long‐acting insulin, either glargine U‐300 or degludec U‐100 can be considered to be initiated first, depending on availability, acceptability, and affordability.
**Recommendation 2 (Conditional Recommendation)**
The initial dose can be considered from 0.10 to 0.20 U/kg/day for any of five basal insulins. For people with impaired kidney function or renal insufficiency, and elderly people over 65 years, dosage for any basal insulin should be reduced as appropriate.
**Recommendation 3 (Conditional Recommendation)**
The maintenance dose can be considered from 0.3 to 0.5 U/kg/day for any of the five basal insulin regimens. In clinical practice, clinicians and patients should use independent medical judgment in the context of individual clinical circumstances.
**Recommendation 4 (Conditional Recommendation)**
The range of controlled FPG of 3.9–6.1 mmol/L (70–110 mg/dL) can be considered for any of the five basal insulin regimens to achieve the ideal HbA_1c_ level in adult patients with T2DM who require basal insulin therapy as clinically indicated. For individuals with a high risk of hypoglycemia or individuals with low requirements for blood sugar control, such as severely ill patients, or individuals with a short life expectancy, 3.9–7.0 mmol/L (70–126 mg/dL) or 4.4–7.0 mmol/L (80–126 mg/dL) of FPG may be considered as clinically indicated. More high‐quality clinical trials are required to investigate other targeted ranges of controlled FPG.
**Qualifying Statement for Recommendation 4**
Individuals with a high risk of hypoglycemia can be defined as patients who have experienced hypoglycemic events or repeated hypoglycemia or are judged by a doctor, the elderly over 65 years, individuals with comorbidities, or the frailty population. Undetectable hypoglycemia has not been evaluated by continuous glucose monitoring in clinical RCTs based on the recommended range of 3.9–6.1 mmol/L (70–110 mg/dL) to control FPG.

Abbreviations: FPG, fasting plasma glucose; HbA_1c_, glycated hemoglobin; NPH, insulin protamine Hagedorn; RCT, randomized controlled trial; T2DM, type 2 diabetes mellitus.

### 3.4 Recommendations, key evidence, and justification

As a point of caution, when we compare insulin A versus (vs.) insulin B for any outcome, insulin A is always treated as the numerator and insulin B as the denominator in the GRADEpro software.^13^ Thus, for insulin A, a favored result of the desirable outcomes is shown as risk ratio (RR) >1, and an undesirable outcome is shown as RR <1. After comparison, all the point estimates crossed at least the small effect threshold are listed in Appendix Table S7.

### 3.5 Q1. What are the differences in the effectiveness and safety among the five basal insulin preparations after the initiation of insulin therapy in adult patients with T2DM?

#### 3.5.1 One insulin bedtime injection versus another insulin bedtime injection

##### Glargine U‐300 versus degludec U‐100

###### Key evidence

Appendix Table S5: The BRIGHT trial with 924 intention‐to‐treat patients met our preplanned study section criteria.^14^, ^15^ It was a multi‐center randomized controlled trial (RCT) carried out in the United States, Bulgaria, Croatia, Czechia, Denmark, France, Greece, Hungary, Israel, Italy, Romania, Serbia, Slovakia, Sweden, Switzerland, and the United Kingdom. Two papers reported subgroup analyses by age (<65 years versus ≥65 years)^16^ and by renal function (<60, ≥60 but <90, ≥90 mL/min/1.73 m^2^),^17^ respectively.

###### Justification

The overall certainty of evidence was low because based on the GRADE handbook, for one comparison, if the evidence of one critical outcome has a low certainty, the overall certainty of evidence is low.^3^ Comparing with degludec U‐100, glargine U‐300 might lead to slightly more patients reaching HbA_1c_ < 7.0% at 6 months and fewer hypoglycemic events at 3 and 6 months but more nocturnal hypoglycemia <3.9 mmol/L at 3 months. The results of other critical outcomes at 3 or 6 months are similar between two groups, such as HbA_1c_ change and nocturnal hypoglycemia<3.0 mmol/L at 3 months; severe hypoglycemia, nocturnal hypoglycemia<3.0 mmol/L, and nocturnal hypoglycemia<3.9 mmol/L at 6 months, etc. One subgroup analysis showed that patients <65 years old or ≥ 65 years old might have consistent results with the overall patients. Another subgroup analysis (n = 365) showed that for patients with estimated glomerular filtration rate (eGFR) of 60–90 mL/min/1.73m^2^, glargine U‐300 might result in more nocturnal hypoglycemia events but fewer hypoglycemia events (<3.0 mmol/L); for patients with eGFR of <60 mL/min/1.73m^2^, glargine U‐300 demonstrated greater HbA_1c_ reduction with potential more nocturnal hypoglycemia (<3.0 mmol/L) at 6 months. However, we do not make recommendations based on subgroup analysis from one study. We call on more high‐quality future research to clarify this point. The prices for both insulins are similar in some countries but may be different in other countries. Additionally, they are unavailable in some areas of certain countries. We did not find evidence for cost‐effectiveness. Based on eligible evidence and clinical experience, we made the following recommendation:

**Table:** 

**Recommendation 1.5 (Conditional Recommendation)**
For ultra‐long‐acting insulin, either glargine U‐300 or degludec U‐100 can be considered to be initiated first, depending on availability, acceptability, and affordability.

##### Glargine U‐100 versus detemir

###### Key evidence

Appendix Table S5: Four RCTs with 1119 patients met our preplanned study section criteria. One study was a multicenter study carried out in Europe and the United States.^18^ Another study was carried out in Canada.^19^ The third one was carried out in Turkey but did not report its timing.^20^ The fourth one was a multicenter study carried out in Argentina, India, Republic of Korea, Thailand, and the United States.^21^

###### Justification

The overall certainty of the evidence was low. Compared with glargine U‐100, detemir probably results in fewer patients having hypoglycemia<3.0 mmol/L at 6 and 12 months (moderate effects), but slightly more patients having nocturnal hypoglycemia<3.0 mmol/L at 6 months (small effect) and fewer patients achieving HbA_1c_ < 7% at 6 and 12 months (large and small effects respectively).^18^, ^21^ In the Rosenstock 2008 RCT 55% of patients received 2 injections at the end of the study in the detemir group.^18^ We did not find evidence for cost‐effectiveness. If these two insulins are available, it would not cause an equity issue. From the patient's point of view, it is more favorable and cost‐efficient to inject insulin once daily compared with twice daily. Based on eligible evidence and clinical experience, we made the following recommendation.

**Table:** 

**Recommendation 1.4 (Conditional Recommendation)**
For long‐acting insulin, if both glargine U‐100 and detemir are available, Glargine U‐100 at bedtime injection can be considered to be initiated first for patients who prefer achieving a HbA_1c_ < 7% during the same period, but may need to pay more attention to potential hypoglycemia. Detemir at bedtime injection can be considered to be initiated first for patients who have concerns about hypoglycemia, but a higher dose and often twice‐a‐day injection may be required.

##### Degludec U‐100 versus glargine U‐100

###### Key evidence

Appendix Table S5: Three RCTs with 2298 patients met our preplanned study section criteria.^22^, ^23^, ^24^ All threestudies were multicenter studies carried out in Hong Kong, Japan, Malaysia, South Korea, Taiwan, and Thailand^22^; Brazil, Canada, China, South Africa, Ukraine, and the United States^23^; and Austria, Belgium, Canada, Czech Republic, Denmark, Finland, France, Germany, Norway, Serbia and Montenegro, Spain, and the United States,^24^ respectively.

##### Glargine U‐300 versus glargine U‐100

###### Key evidence

Appendix Table S5: The Comparison of a New Formulation of Insulin Glargine With Lantus in Patients With Type 2 Diabetes on Non‐insulin Antidiabetic Therapy (EDITION 3 trial) with 878 patients was a multicenter study carried out in North America, Europe, and Japan,^25^, ^26^ and another RCT with 604 patients met our preplanned study section criteria conducted in China.^27^

###### Justification

The overall certainty of the evidence was low. Compared with glargine U‐100, degludec U‐100 may lead to less severe hypoglycemia (moderate effect) and nocturnal hypoglycemia (small effect); Glargine U‐300 may lead to less hypoglycemia (large effect) and nocturnal hypoglycemia (large effect), with more patients achieving HbA_1c_<7.0%. However, when generic degludec U‐100 or glargine U‐300 is unavailable, both glargine U‐300 and degludec U‐100 are more expensive than glargine U‐100. Thus, it may affect equity and acceptability. Based on eligible evidence and clinical experience, we made the following recommendation.

**Table:** 

**Recommendation 1.2 (Strong Recommendation)**
If both ultra‐long‐acting and long‐acting insulin regimens are available, ultra‐long‐acting insulins (glargine U‐300 and degludec U‐100) at bedtime injection should be considered to be initiated first depending on acceptability and affordability.

##### Glargine U‐300 versus NPH

###### Key evidence

Appendix Table S5: One RCT with 50 patients met our preplanned study section criteria,^28^ which was carried out in China.

##### Glargine U‐100 versus NPH

###### Key evidence

Appendix Table S5: Twelve RCTs with 4233 patients met our preplanned study section criteria.^29^, ^30^, ^31^, ^32^, ^33^, ^34^, ^35^, ^36^, ^37^, ^38^, ^39^, ^40^ One study was carried out in the United States.^38^ The other eight studies were part of a multicenter study carried out in Finland and the United Kingdom^39^; China, Hong Kong, Indonesia, South Korea, Malaysia, Pakistan, Philippines, Taiwan, Thailand and Singapore^36^; the United States, and Canada^37^; European countries^31^; Argentina, Brazil, Chile, Colombia, Guatemala, Mexico, Paraguay, Peru, Uruguay, and Venezuela^29^; European countries and South Africa^34^; Germany and Finland^40^; and Europe (nine sites), Asia (three sites), the Middle East (two sites), and South America (two sites)^33^ respectively.

##### Detemir versus NPH

###### Key evidence

Appendix Table S5: One RCT with 333 patients met our preplanned study section criteria, which was a multicenter study carried out in Denmark, France, Italy, The Netherlands, Norway, Spain, and the United States.^41^

###### Justification

The overall certainty of evidence was low. Compared with NPH, glargine U‐300 or detemir may result in a large reduction in hypoglycemia and nocturnal hypoglycemia; glargine U‐100 may result in an increase in patients achieving HbA_1c_ < 7%, a reduction in hypoglycemia, and a large reduction in nocturnal hypoglycemia. There is no eligible evidence to compare degludec U‐100 with NPH. From the indirect evidence (network meta‐analyses) between degludec U‐100 and glargine U‐100,^7^ it may be reasonable to conclude that degludec U‐100 can lead to better clinical outcomes than NPH overall. We did not find evidence for cost‐effectiveness. However, the price of glargine U‐300, degludec U‐100, glargine U‐100, and detemir is more expensive than that of NPH. Thus, it may affect equity and patients' acceptability. Based on eligible evidence and clinical experience, we made the following recommendation.

**Table:** 

**Recommendation 1.1 (Strong Recommendation)**
Among the five basal insulins, we recommend choosing any of glargine U‐300, degludec U‐100, glargine U‐100, and detemir over NPH at bedtime injection, depending on availability, acceptability, and affordability.
**Qualifying Statement for Recommendation 1.1**
The current recommendation is based on the protocol of eligible RCTs, in which the injection of the investigated insulins is administrated at bedtime. There is insufficient direct evidence to compare the effectiveness and safety among glargine U‐300, degludec U‐100, glargine U‐100, and detemir injected in the morning; there is no eligible evidence to compare morning versus bedtime injection for glargine U‐300 or degludec U‐100 individually in T2DM patients who initially injected basal insulins. Future high‐quality RCTs are required to solve these issues.

#### 3.5.2 One insulin morning versus another insulin morning injection

##### Degludec U‐100 morning versus glargine U‐100 morning time

###### Key evidence

Appendix Table S5: One RCT with 44 patients met our preplanned study section criteria.^42^ It was a multicenter study carried out in Japan.

###### Justification

The overall certainty of evidence was low. Because this eligible RCT had a sample size of only 44 (one group had 32 patients and another group had 12 patients), we do not make a recommendation based on the results of this trial.

##### Detemir versus NPH

###### Key evidence

Appendix Table S5: One RCT with 86 patients met our preplanned study section criteria,^43^ which was a multicenter study carried out in France, and the United Kingdom.^43^

###### Justification

The overall certainty of the evidence was low. This paper was not a peer‐reviewed journal publication, and it was published as a pharmaceutical report with no authors indicated. Thus, we do not make a recommendation based on it.

#### 3.5.3 One insulin morning injection versus another insulin bedtime injection

##### Detemir morning time versus NPH bedtime

###### Key evidence

Appendix Table S5: One RCT with 329 patients met our preplanned study section criteria, which was a multicenter study conducted in Denmark, France, Italy, The Netherlands, Norway, Spain, and the United States.^41^

###### Justification

The overall certainty of the evidence was low. The results are consistent with that under 3.5.1 when detemir bedtime injection was compared with NPH bedtime injection. That is, detemir may result in better hypoglycemia and nocturnal hypoglycemia outcomes. Therefore, we do not make an additional recommendation.

##### Glargine U‐100 morning time versus NPH bedtime

###### Key evidence

Appendix Table S5: Two RCTs with 526 patients met our preplanned study section criteria.^31^, ^38^ One study was a multicenter study carried out in European countries.^31^ Another study was conducted in the United States.^38^

###### Justification

The overall certainty of the evidence was low. The results are consistent with that under 3.5.1 when glargine U‐100 bedtime injection was compared with NPH bedtime injection. That is, glargine U‐100 may result in an increase in patients who achieved HbA_1c_ < 7%, a reduction in hypoglycemia. Hence, we do not make an additional recommendation.

#### 3.5.4 Same insulin morning versus bedtime injection

##### Detemir morning time versus detemir bedtime

###### Key evidence

Appendix Table S5: One RCT with 334 patients met study section criteria, which was a multicenter study carried out in Denmark, France, Italy, The Netherlands, Norway, Spain, and the United States.^41^

###### Justification

The overall certainty of the evidence was low. Based on the above magnitude of effects, there is insufficient evidence to make a recommendation for this comparison.

##### Glargine U‐100 morning time versus glargine U‐100 bedtime

###### Key evidence

Appendix Table S5: Two RCTs with 521 patients met our preplanned study section criteria.^31^, ^38^ One study was a multi‐center study carried out in European countries.^31^ Another study was carried out in the United States.^38^

###### Justification

The overall certainty of the evidence was low. For glargine U‐100, morning injection may increase the number of patients who achieved HbA_1c_ < 7% and reduce nocturnal hypoglycemia events. Thus, if patients can accept morning injection, it may be a better choice. Based on eligible evidence and clinical experience, we made the following recommendation.

**Table:** 

**Recommendation 1.3 (Conditional Recommendation)**
Glargine U‐100 can be administered either in the morning or at bedtime. Injection in the morning can be considered first depending on patients' acceptability.

### 3.6 Q2. What are the optimal starting doses (U/kg/day) and time of administration (morning versus bedtime) of five basal insulins?

#### 3.6.1 Key evidence

There is no eligible RCT to compare the different initial doses for glargine U‐300, degludec U‐100, or NPH to control blood glucose with acceptable side effects for adult patients with T2DM who require basal insulin therapy. Among the 35 included RCTs in our systematic review, eight RCTs indicated that the initial dose for glargine U‐300 was 0.20 U/kg/day,^14^, ^15^, ^16^, ^17^, ^25^, ^26^, ^27^, ^28^ for degludec U‐100 was from 0.10 to 0.20 U/kg/day,^14^, ^15^, ^16^, ^17^, ^22^, ^23^, ^24^, ^42^ and for NPH was from 0.11 to 0.23 U/kg/day^28^, ^31^, ^32^, ^33^, ^36^, ^38^, ^39^, ^43^

One RCT with 60 patients carried out in Turkey compared the initial dose of 0.12 U/kg/day once daily with 0.12 U/kg/day twice daily for detemir.^44^ Based on the results, we do not feel confident to recommend either strategy.^7^ Among the 35 included RCTs in our systematic review, six RCTs indicated the range of the initial dose for detemir was from 0.10 to 0.20 U/kg/day.^18^, ^19^, ^20^, ^21^, ^43^, ^45^

One RCT with 892 patients carried out in China compared the initial dose of glargine U‐100 0.2 U/kg/day with glargine U‐100 0.3 U/kg/day.^46^ The results did not favor U‐100 0.3 U/kg/day over 0.2 U/kg/day to lead to better patient‐related outcomes. Among the 35 included RCTs, 17 RCTs indicated that the range of the initial dose for glargine U‐100 was from 0.10 to 0.20 U/kg/day.^18^, ^19^, ^20^, ^21^, ^22^, ^23^, ^24^, ^25^, ^26^, ^32^, ^33^, ^36^, ^38^, ^39^, ^42^, ^47^, ^48^

#### 3.6.2 Justification

The working group members made the following consensus conditional recommendation based on the data from eligible publications and clinical experience. Considering the simplicity of practical applications, we suggested the same range of initial doses for any of the five basal insulins. In clinical practice, clinicians and patients should use independent medical judgment in the context of individual clinical circumstances.

**Table:** 

**Recommendation 2 (Conditional Recommendation)**
The initial dose can be considered from 0.10 to 0.20 U/kg/day for any of five basal insulins. For people with impaired kidney function or renal insufficiency, and the elderly people over 65 years, dosage for any basal insulin should be reduced as appropriate.

### 3.7 Q3. Among the target population treated with any one of the five basal insulin preparations, what is the optimal maintenance dose (U/kg/day) that achieves target fasting plasma glucose?

#### 3.7.1 Key evidence

There is no eligible RCT to compare the different initial doses for any of five insulins to lead to a satisfactory control of FPG in adult patients with T2DM who require basal insulin therapy. Among the 35 included RCTs in our systematic review, seven RCTs indicated that the range of the end point dose for glargine U‐300 was from 0.34 to 0.62 U/kg/day at bedtime administration^14^, ^15^, ^16^, ^17^, ^25^, ^26^, ^27^; seven RCTs indicated the range of the end point dose for degludec U‐100 was from 0.28 to 0.59 U/kg/day at bedtime administration^14^, ^15^, ^16^, ^17^, ^22^, ^23^, ^24^; seven RCTs indicated the range for detemir was from 0.19 to 0.78 U/kg/day at bedtime administration^18^, ^19^, ^20^, ^21^, ^41^, ^44^, ^45^; 20 RCTs that indicated the range for glargine U‐100 was from 0.34 to 0.62 U/kg/day^18^, ^19^, ^20^, ^21^, ^22^, ^23^, ^24^, ^25^, ^26^, ^27^, ^31^, ^32^, ^33^, ^37^, ^38^, ^39^, ^40^, ^46^, ^47^, ^48^; eight RCTs that indicated the range of the end point dose for NPH was from 0.19 to 0.66 U/kg/day at bedtime administration^31^, ^32^, ^33^, ^37^, ^38^, ^39^, ^40^, ^41^

#### 3.7.2 Justification

There is insufficient evidence regarding the optimal maintenance dose range that can lead to a satisfactory control of FPG. Factors associated with the decision of the initial dose in addition to age and renal function (eg, body weight and baseline glycemic control condition, such as FPG and HbA_1c_) should also be taken into consideration. The 2022 American Diabetes Association guideline stated that when the basal dose is more than 0.5 U/kg/day, overbasalization needs to be evaluated.^2^ Another study that pooled and analyzed patient‐level data from 15 RCTs in patients with T2DM treated with insulin glargine indicated that increasing basal insulin doses above 0.5 IU/kg was not associated with improvement in glycemic control.^49^ Thus, the working group members made the following conditional recommendations based on the data from eligible publications and clinical experience. Considering the simplicity of practical applications, we suggested the same range of initial doses for any of the five basal insulin regimens. The individualized approach according to the patient's clinical circumstances should be considered thoroughly.

**Table:** 

**Recommendation 3 (Conditional Recommendation)**
The maintenance dose can be considered from 0.3 to 0.5 U/kg/day for any of the five basal insulin regimens. In clinical practice, clinicians and patients should use independent medical judgment in the context of individual clinical circumstances.

### 3.8 Q4. After initiation of any of the five basal insulins, what range of the target FPG can lead to the ideal HbA_1c_ level (ie, <7.0%) in the target population?

#### 3.8.1 Key evidence

Appendix Table S8: Three RCTs met our preplanned study section criteria.^45^, ^47^, ^48^ One RCT with 244 patients investigated detemir and was conducted in the United States.^45^ Two other RCTs with 1018 patients focused on glargine U‐100 and were conducted in China.^47^, ^48^ There is no eligible RCT to answer this research question for glargine U‐300, degludec U‐100, or NPH.

#### 3.8.2 Justification

The overall certainty of the evidence was low. For glargine U‐100, compared with 3.9 < FPG≤5.6 mmol/L, 3.9 < FPG≤6.1 mmol/L may result in more patients achieving HbA_1c_ < 7% and fewer patients experiencing hypoglycemia and nocturnal hypoglycemia; compared with 3.9 < FPG≤7.0 mmol/L, 3.9 < FPG≤6.1 mmol/L may result in more patients achieving HbA_1c_ < 7% (moderate effect) without a big tradeoff of patients who experienced hypoglycemia (trivial effect) and nocturnal hypoglycemia (small effect). Controlling hyperglycemia and preventing hypoglycemia are always tradeoffs. Thus, whether raising the lower boundary of the target FPG can reduce hypoglycemia risk without compromising glycemic control should be further investigated. Although this evidence is from glargine U‐100, the working group members believe it can be applied to any four basal insulin regimens. The 2022 American Diabetes Association guideline stated that the expected range of time in range is from 3.9 to 10 mmol/L for continuous glucose monitoring.^50^ Hence, we made the following recommendation based on limited evidence, their clinical experience, and external reviewers' comments.

**Table:** 

**Recommendation 4 (Conditional Recommendation)**
The range of controlled FPG of 3.9–6.1 mmol/L (70–110 mg/dL) can be considered for any of the five basal insulin regimens to achieve the ideal HbA_1c_ level in adult patients with T2DM who require basal insulin therapy as clinically indicated. For individuals with a high risk of hypoglycemia or individuals with low requirements for blood sugar control, such as severely ill patients or individuals with a short life expectancy, 3.9–7.0 mmol/L (70–126 mg/dL) or 4.4–7.0 mmol/L (80–126 mg/dL) of FPG may be considered as clinically indicated. More high‐quality clinical trials are required to investigate other targeted ranges of controlled FPG.
**Qualifying Statement for Recommendation 4**
Individuals with a high risk of hypoglycemia can be defined as patients who have experienced hypoglycemic events or repeated hypoglycemia or are judged by a doctor, elderly people over 65 years, individuals with comorbidities, or the frailty population. Undetectable hypoglycemia has not been evaluated by continuous glucose monitoring in clinical RCTs based on the recommended range of 3.9–6.1 mmol/L (70–110 mg/dL) to control FPG.

### 3.9 Recommendations summary

We summarized these recommendations in Table 2 and reordered the sequence of the recommendations for end users to easily find and implement.

## 4 DISCUSSION AND FUTURE RESEARCH

As we had summarized from the available clinical evidence, compared with NPH, in addition to longer long‐acting time, basal insulin analogs also have more flattened pharmacokinetic and pharmacodynamic profiles that are attributable to less hypoglycemic risk, especially nocturnal hypoglycemia. But because all basal insulin analogs have peaks and troughs in their blood glucose lowering effects, the hypoglycemic risk of that insulin preparation might be different if the insulin were administered at a different time of the day (ie, morning or bedtime). Although we found 35 eligible papers after a systematic literature search, to date, there is insufficient evidence to conduct a direct comparison between detemir and glargine U‐300 or degludec U‐100 at bedtime. Additionally, there is insufficient direct evidence to compare the effectiveness and safety among glargine U‐300, degludec U‐100, glargine U‐100, and detemir injected in the morning. Although some researchers indicated that the mean 24‐h glucose curves were smooth for the glargine U‐300 group irrespective of morning or evening injection in patients with T1DM,^51^ there is no eligible evidence comparing morning versus bedtime injection for glargine U‐300 or degludec U‐100 individually in T2DM patients who initially injected basal insulins. Furthermore, there is no paper among the 35 papers discussing overbasalization. Seven papers (7 RCTs) recruited only Asian‐Pacific patients; 6 papers (5 RCTs) mixed Asian‐Pacific patients but without subgroup analyses for Asian patients; 22 papers (19 RCTs) did not include Asian‐Pacific patients. The studies conducted in the non‐Asia‐Pacific region, recruited some patients who came from the Asia‐Pacific region; however, there was no subgroup analysis for Asian‐Pacific patients. Because the 16 clinical experts from working group members for this guideline came from nine countries in the Asia‐Pacific region and the 34 external reviewers from nine countries, and the initial doses and maintenance doses were reported in units/kilogram/day from the original studies, the recommendations of this guideline can be implemented in any country in the Asia‐Pacific region and even in the world if the basal insulins are available.

It should be noted that clinical significance is different from statistical significance.^52^ For example, when glargine U‐300 was compared with glargine U‐100, there were 54 more patients/1000 that reached HbA_1c_ < 7.0% at 12 months (95% confidence interval [CI], 5 fewer to 127 more) in the glargine U‐300 group. Because the point estimate of 54 is higher than our clinical threshold of the moderate effect (50 patients/1000), its upper boundary of 95% CI was 127, which was higher than the clinical threshold of the large effect (>100 patients/1000), and its lower boundary of the 95% CI was 5 fewer, which was lower than the clinical threshold of small effect (30 patients/1000) to favor glargine U‐100, we can consider that it may be clinically significant favoring degludec U‐100 even though it was not statistically significant. A few external reviewers disagreed with our recommendations largely because they considered statistical significance rather than clinical significance for the result of an outcome. Thus, setting up clinical thresholds is a very important step in developing clinical practice guidelines. This point is emphasized in the current GRADE publication.^9^ In the diabetes community, this is the first guideline to set up clinical thresholds of trivial, small, moderate, and large effects for critical and important outcomes, which can be regarded as references for future researchers to consider and modify according to their individual contexts if needed.

In clinical practice, every patient has his/her own individual conditions (such as age, weight, duration of diabetes, morbidities, and accessibility of insulins). Thus, the recommendations and qualifying statements in this guideline should be used only as references. Clinicians should make the appropriate modifications when implementing them for each patient. Additionally, when the evidence with continuous glucose monitoring is available, these recommendations may be changed.

We understand that with low certainty of evidence, different working group members may make different recommendations regarding wording and strength based on their context. Thus, keeping the process of developing this guideline transparent, we hope that users will more easily be able to implement any of the recommendations in their daily clinical practice based on their individual contexts. Future high‐quality research is called to investigate the issues discussed and fill gaps in knowledge.

## 5 GUIDELINE LIMITATIONS

First, the cost‐effectiveness of the five basal insulins was not assessed because of the lack of evidence that met our study selection criteria. In addition, different countries may need the country‐specific cost‐efficacy analysis owing to the availability of these five basal insulins. Second, we did not recruit patient representatives in the working group and found only one patient representative as an external reviewer due to the time limitation. Thus, the patients' values may not be reflected in our recommendations sufficiently. Third, due to the time limitation, the working group was unable to include several large countries in the Asia‐Pacific region, such as India, the Philippines, etc., and were unable to get more external reviewers to complete the online survey, which may limit this study's generalizability.

## 6 UPDATING

When new evidence changes any of the recommendations, this guideline will be updated by the authors as soon as possible.

## AUTHOR CONTRIBUTIONS

Linong Ji, Yingying Luo, Jun Xia, and Xiaomei Yao conceived and designed this study. Zhan Zhao and Chenchen Xu conducted the database search and reviewed the reference lists of articles included in the screening. Zhan Zhao and Chenchen Xuperformed the initial screening and review of full texts for eligibility and inclusion. Zhan Zhao and Chenchen Xuextracted the data and completed the quality assessments. Xiaomei Yaoresolved any conflicts in the quality assessments. Zhan Zhao prepared the tables, and conducted the data analysis. Linong Ji, Xiaomei Yao, and Yingying Luo conducted data interpretation, led group discussions, and drafted the first draft of the manuscript. All authors approved the project plan and reviewed and revised the final manuscript before submission.

## FUNDING INFORMATION

Sponsored by the Chinese Geriatric Endocrine Society. The Chinese Geriatric Endocrine Society is a not‐for‐profit organization that accepts donations and support from communities and industries. All work done by the authors is editorially independent of the Chinese Geriatric Endocrine Society.

## CONFLICT OF INTEREST STATEMENT

Within the past 4 years, Yong Mong Bee and Daisuke Yabe have received consulting remuneration from a commercial entity or other organization with an interest related to subjectiveness of the meeting or work; SiewPheng Chan, Margaret McGill, Daisuke Yabe, or their research teams have received support from a commercial entity or other organization with an interest related to subjectiveness of the meeting or work respectively; Siew Pheng Chan also has received nonmonetary support valued at more than US $1000 (including equipment, facilities, research assistants, paid travel meetings, etc.). Ketut Suastika received an honorarium for a scientific symposium or webinar on basal insulin from several pharmaceutical companies, which were events collaborated with the Indonesia Society of Endocrinology. Alice Pik Shan Kong received research grants and/or speaker honoraria from Abbott, Astra Zeneca, Bayer, Boehringer Ingelheim, Eli‐Lilly, Kyowa Kirin, Merck Serono, Nestle, Novo‐Nordisk, Pfizer, and Sanofi. Khue Nguyen Thy received an honorarium for the chair in scientific meetings from Servier, Boehringer Ingelheim, and Eisai. Linong Ji has received consulting and lecture fees from Eli Lilly, Novo Nordisk, Merck, Bayer, Sanofi‐Aventis, Roche, MSD, Metronics AstraZeneca, Boehinger Ingelheim, and Abbott. Other authors declare no competing interest.

## Supporting information


**Appendix S1.** Supplementary Information.

## Data Availability

Most of the data are available in supplements. Additional requests can be provided by contacting authors.

## References

[1] Chan SP , Aamir AH , Bee YM , et al. Practical guidance on basal insulin initiation and titration in Asia: a Delphi‐based consensus. Diabetes Ther. 2022;13(8):1511‐1529. 35767186 10.1007/s13300-022-01286-0 PMC9309111

[2] American Diabetes Association Professional Practice Committee , Draznin B , Aroda VR , et al. Pharmacologic approaches to glycemic treatment: standards of medical Care in Diabetes‐2022. Diabetes Care. 2022;45(Suppl 1):S125‐S143. 34964831 10.2337/dc22-S009

[3] Schünemann H , Brozek J , Guyatt G , Oxman A . Handbook for grading the quality of evidence and the strength of recommendations using the GRADE approach. 2013. https://gdt.gradepro.org/app/handbook/handbook.html Accessed on Dec 30 2022.

[4] Brouwers MC , Kerkvliet K , Spithoff K , AGREE Next Steps Consortium . The AGREE reporting checklist: a tool to improve reporting of clinical practice guidelines. BMJ. 2016;352:i1152. 26957104 10.1136/bmj.i1152 PMC5118873

[5] Chen Y , Yang K , Marusic A , et al. Ein instrument zur Erstellung von Leitlinienberichten: das RIGHT‐statement [a reporting tool for practice guidelines in health care: the RIGHT statement]. Z Evid Fortbild Qual Gesundhwes. 2017;127‐128:3‐10. 10.1016/j.zefq.2017.10.008 29128430

[6] Yao X , Ma J , Wag Q , Kanters D , Usman A , Florez I . A comparison of AGREE and RIGHT: which clinical practice guideline reporting checklist should Be followed by guideline developers? J Gen Intern Med. 2020;35:894‐898. 31713037 10.1007/s11606-019-05508-3 PMC7080917

[7] Luo Y , Xia J , Zhao Z , et al. Effectiveness, safety, initial optimal dose, and optimal maintenance dose range of basal insulin regimens for type 2 diabetes: A systematic review with meta‐analysis. J Diabetes. Epub April 10, 2023. doi: 10.1111/1753-0407.13381 PMC10172019 37038616

[8] Basal Insulin Initiation and Dose Titration in Adults with Type 2 Diabetes: an Asia‐Pacific Clinical Practice Guideline. https://guidelinesebmportalcom/node/70108 Accessed 27 December 2022.

[9] Schünemann HJ , Neumann I , Hultcrantz M , et al. GRADE guidance 35: update on rating imprecision for assessing contextualized certainty of evidence and making decisions. J Clin Epidemiol. 2022;150:225‐242. 35934266 10.1016/j.jclinepi.2022.07.015

[10] Little RR , Rohlfing CL . The long and winding road to optimal HbA_1c_ measurement. Clin Chim Acta. 2013;418:63‐71. 23318564 10.1016/j.cca.2012.12.026 PMC4762213

[11] Lisi DM . Applying recent A1C recommendations in clinical practice. US Pharm. 2018;43(10):15‐22.

[12] Yao X , Ghert M , Dickson BC , et al. An evidence‐based guideline on the application of molecular testing in the diagnosis, prediction of prognosis, and selection of therapy in non‐GIST soft tissue sarcomas. Cancer Treat Rev. 2020;85:101987. 32092619 10.1016/j.ctrv.2020.101987

[13] GRADEpro software. https://gdtgradeproorg/app/ Accessed 27 December 2022.

[14] Cheng A , Harris S , Giorgino F , et al. Similar glycaemic control and less hypoglycaemia during active titration after insulin initiation with glargine 300 units/mL and degludec 100 units/mL: a subanalysis of the BRIGHT study. Diabetes Obes Metab. 2020;22(3):346‐354. 31646724 10.1111/dom.13901 PMC7064957

[15] Rosenstock J , Cheng A , Ritzel R , et al. More similarities than differences testing insulin glargine 300 units/mL versus insulin Degludec 100 units/mL in insulin‐naive type 2 diabetes: the randomized head‐to‐head BRIGHT trial. Diabetes Care. 2018;41(10):2147‐2154. 30104294 10.2337/dc18-0559

[16] Bolli GB , Cheng A , Charbonnel B , et al. Glycaemic control and hypoglycaemia risk with insulin glargine 300 U/mL and insulin degludec 100 U/mL in older participants in the BRIGHT trial. Diabetes Obes Metab. 2021;23(7):1588‐1593. 33687748 10.1111/dom.14372 PMC8252805

[17] Haluzík M , Cheng A , Müller‐Wieland D , et al. Differential glycaemic control with basal insulin glargine 300 U/mL versus degludec 100 U/mL according to kidney function in type 2 diabetes: a subanalysis from the BRIGHT trial. Diabetes Obes Metab. 2020;22(8):1369‐1377. 32243043 10.1111/dom.14043 PMC7383874

[18] Rosenstock J , Davies M , Home PD , Larsen J , Koenen C , Schernthaner G . A randomised, 52‐week, treat‐to‐target trial comparing insulin detemir with insulin glargine when administered as add‐on to glucose‐lowering drugs in insulin‐naive people with type 2 diabetes. Diabetologia. 2008;51(3):408‐416. 18204830 10.1007/s00125-007-0911-x PMC2235909

[19] Elisha B , Azar M , Taleb N , Bernard S , Iacobellis G , Rabasa‐Lhoret R . Body composition and Epicardial fat in type 2 diabetes patients following insulin Detemir versus insulin glargine initiation. Horm Metab Res. 2015;48(1):42‐47. 26340704 10.1055/s-0035-1554688

[20] Cander S , Gül ÖÖ , Dizdar OS , Aydın T , Ersoy C . The effect of basal analog insulin on the glycemic variability in type 2 diabetics. Turk J Endocrinol Metab. 2014;2:33‐38.

[21] Meneghini LKJ , Demissie M , Nazeri A , Hollander P . Once‐daily initiation of basal insulin as add‐on to metformin: a 26‐week, randomized, treat‐to‐target trial comparing insulin detemir with insulin glargine in patients with type 2 diabetes. Diabetes Obes Metab. 2013;15(8):729‐736. 23421331 10.1111/dom.12083

[22] Onishi Y , Iwamoto Y , Yoo SJ , Clauson P , Tamer SC , Park S . Insulin degludec compared with insulin glargine in insulin‐naïve patients with type 2 diabetes: a 26‐week, randomized, controlled, Pan‐Asian, treat‐to‐target trial. J Diabetes Investig. 2013;4(6):605‐612. 10.1111/jdi.12102 PMC4020256 24843715

[23] Pan C , Gross JL , Yang W , et al. A multinational, randomized, open‐label, treat‐to‐target trial comparing insulin Degludec and insulin glargine in insulin‐Naïve patients with type 2 diabetes mellitus. Drugs R D. 2016;16(2):239‐249. 27098525 10.1007/s40268-016-0134-z PMC4875929

[24] Zinman B , Philis‐Tsimikas A , Cariou B , et al. Insulin degludec versus insulin glargine in insulin‐naive patients with type 2 diabetes: a 1‐year, randomized, treat‐to‐target trial (BEGIN once long). Diabetes Care. 2012;35(12):2464‐2471. 23043166 10.2337/dc12-1205 PMC3507614

[25] Bolli GB , Riddle MC , Bergenstal RM , et al. New insulin glargine 300 U/ml compared with glargine 100 U/ml in insulin‐naïve people with type 2 diabetes on oral glucose‐lowering drugs: a randomized controlled trial (EDITION 3). Diabetes Obes Metab. 2015;17(4):386‐394. 25641260 10.1111/dom.12438 PMC4409854

[26] Bolli GB , Riddle MC , Bergenstal RM , Wardecki M , Goyeau H , Home PD . Glycaemic control and hypoglycaemia with insulin glargine 300U/mL versus insulin glargine 100U/mL in insulin‐naïve people with type 2 diabetes: 12‐month results from the EDITION 3 trial. Diabetes Metab. 2017;43(4):351‐358. 28622950 10.1016/j.diabet.2017.04.007

[27] Ji L , Kang ES , Dong X , et al. Efficacy and safety of insulin glargine 300 U/mL versus insulin glargine 100 U/mL in Asia Pacific insulin‐naïve people with type 2 diabetes: the EDITION AP randomized controlled trial. Diabetes Obes Metab. 2020;22(4):612‐621. 31797549 10.1111/dom.13936 PMC7384042

[28] Ling J , Poon EWM , Yang A , et al. Glycemic variability and time in range during self‐titration of once daily insulin glargine 300 U/ml versus neutral protamine Hagedorn insulin in insulin‐naïve Chinese type 2 diabetes patients. Diabetes Ther. 2021;12(5):1399‐1413. 33738774 10.1007/s13300-021-01046-6 PMC8099948

[29] Eliaschewitz FG , Calvo C , Valbuena H , et al. Therapy in type 2 diabetes: insulin glargine vs. NPH insulin both in combination with glimepiride. Arch Med Res. 2006;37(4):495‐501. 16715577 10.1016/j.arcmed.2005.10.015

[30] Forst T , Larbig M , Hohberg C , et al. Adding insulin glargine vs. NPH insulin to metformin results in a more efficient postprandial beta‐cell protection in individuals with type 2 diabetes. Diabetes Obes Metab. 2010;12(5):437‐441. 20415692 10.1111/j.1463-1326.2010.01209.x PMC2871167

[31] Fritsche A , Schweitzer MA , Häring HU . Glimepiride combined with morning insulin glargine, bedtime neutral protamine hagedorn insulin, or bedtime insulin glargine in patients with type 2 diabetes. A randomized, controlled trial. Ann Intern Med. 2003;138(12):952‐959. 12809451 10.7326/0003-4819-138-12-200306170-00006

[32] Hermanns N , Kulzer B , Kohlmann T , et al. Treatment satisfaction and quality‐of‐life between type 2 diabetes patients initiating long‐ vs. intermediate‐acting basal insulin therapy in combination with oral hypoglycemic agents—a randomized, prospective, crossover, open clinical trial. Health Qual Life Outcomes. 2015;13:77. 26055391 10.1186/s12955-015-0279-4 PMC4459660

[33] Home PD , Bolli GB , Mathieu C , et al. Modulation of insulin dose titration using a hypoglycaemia‐sensitive algorithm: insulin glargine versus neutral protamine Hagedorn insulin in insulin‐naïve people with type 2 diabetes. Diabetes Obes Metab. 2015;17(1):15‐22. 24957785 10.1111/dom.12329 PMC4282751

[34] Massi Benedetti M , Humburg E , Dressler A , Ziemen M . A one‐year, randomised, multicentre trial comparing insulin glargine with NPH insulin in combination with oral agents in patients with type 2 diabetes. Horm Metab Res. 2003;35(3):189‐196. 12734781 10.1055/s-2003-39080

[35] Oikonomou D , Kopf S , von Bauer R , et al. Influence of insulin and glargine on outgrowth and number of circulating endothelial progenitor cells in type 2 diabetes patients: a partially double‐blind, randomized, three‐arm unicenter study. Cardiovasc Diabetol. 2014;13:137. 25300286 10.1186/s12933-014-0137-4 PMC4195950

[36] Pan CY , Sinnassamy P , Chung KD , Kim KW . Insulin glargine versus NPH insulin therapy in Asian type 2 diabetes patients. Diabetes Res Clin Pract. 2007;76(1):111‐118. 17011662 10.1016/j.diabres.2006.08.012

[37] Riddle MC , Rosenstock J , Gerich J . The treat‐to‐target trial: randomized addition of glargine or human NPH insulin to oral therapy of type 2 diabetic patients. Diabetes Care. 2003;26(11):3080‐3086. 14578243 10.2337/diacare.26.11.3080

[38] Hsia SH . Insulin glargine compared to NPH among insulin‐naïve, U.S. inner city, ethnic minority type 2 diabetic patients. Diabetes Res Clin Pract. 2011;91(3):293‐299. 21146881 10.1016/j.diabres.2010.11.028 PMC3055948

[39] Yki‐Järvinen H , Kauppinen‐Mäkelin R , Tiikkainen M , et al. Insulin glargine or NPH combined with metformin in type 2 diabetes: the LANMET study. Diabetologia. 2006;49(3):442‐451. 16456680 10.1007/s00125-005-0132-0

[40] Yki‐Järvinen H , Dressler A , Ziemen M . Less nocturnal hypoglycemia and better post‐dinner glucose control with bedtime insulin glargine compared with bedtime NPH insulin during insulin combination therapy in type 2 diabetes. HOE 901/3002 study group. Diabetes Care. 2000;23(8):1130‐1136. 10937510 10.2337/diacare.23.8.1130

[41] Philis‐Tsimikas A , Charpentier G , Clauson P , Ravn GM , Roberts VL , Thorsteinsson B . Comparison of once‐daily insulin detemir with NPH insulin added to a regimen of oral antidiabetic drugs in poorly controlled type 2 diabetes. Clin Ther. 2006;28(10):1569‐1581. 17157113 10.1016/j.clinthera.2006.10.020

[42] Aso Y , Suzuki K , Chiba Y , et al. Effect of insulin degludec versus insulin glargine on glycemic control and daily fasting blood glucose variability in insulin‐naïve Japanese patients with type 2 diabetes: I'D GOT trial. Diabetes Res Clin Pract. 2017;130:237‐243. 28651211 10.1016/j.diabres.2017.06.007

[43] Novo Nordisk pharmaceutical company. A seven‐month, open‐labelled, randomised, multicentre, two‐group, parallel trial comparing administration of insulin detemir once daily in the morning versus NPH insulin once daily in ageing patients with type 2 diabetes: the 3L Study (Levemir in Later Life) (NN304‐1808 Clinical Trial Report). http://filehostingpharmacmcom/DownloadServiceashx?client=CTR_NVO_7271&studyid=1426&filename=1808-ctr-redactedpdf 2020.

[44] Cander S , Dizdar OS , Oz Gul O , et al. Comparison of efficacy and safety of once‐ versus twice‐daily insulin detemir added on to oral antidiabetics in insulin‐naive type 2 diabetes patients: 24‐week, crossover, treat to target trial in a single center. Prim Care Diabetes. 2014;8(3):256‐264. 24522170 10.1016/j.pcd.2014.01.010

[45] Blonde L , Merilainen M , Karwe V , Raskin P . Patient‐directed titration for achieving glycaemic goals using a once‐daily basal insulin analogue: an assessment of two different fasting plasma glucose targets—the TITRATE TM study. Diabetes Obes Metab. 2009;11(6):623‐631. 19515182 10.1111/j.1463-1326.2009.01060.x

[46] Ji L , Wan H , Wen B , et al. Higher versus standard starting dose of insulin glargine 100 U/mL in overweight or obese Chinese patients with type 2 diabetes: results of a multicentre, open‐label, randomized controlled trial (BEYOND VII). Diabetes Obes Metab. 2020;22(5):838‐846. 31944546 10.1111/dom.13967 PMC7187195

[47] Yang W , Ma J , Yuan G , et al. Determining the optimal fasting glucose target for patients with type 2 diabetes: results of the multicentre, open‐label, randomized‐controlled FPG GOAL trial. Diabetes Obes Metab. 2019;21(8):1973‐1977. 30938035 10.1111/dom.13733 PMC6772047

[48] Yuan L , Li F , Zhou Y , et al. Fasting glucose of 6.1 mmol/L as a possible optimal target for type 2 diabetic patients with insulin glargine: a randomized clinical trial. J Diabetes Res. 2021;2021:5524313‐5524319. 34337072 10.1155/2021/5524313 PMC8294995

[49] Reid T , Gao L , Gill J , et al. How much is too much? Outcomes in patients using high‐dose insulin glargine. Int J Clin Pract. 2016;70(1):56‐65. 26566714 10.1111/ijcp.12747 PMC4738456

[50] American Diabetes Association Professional Practice Committee , Draznin B , Aroda VR , et al. 6. Glycemic targets: standards of medical Care in Diabetes‐2022. Diabetes Care. 2022;45(Suppl 1):S83‐S96. 34964868 10.2337/dc22-S006

[51] Bergenstal RM , Bailey TS , Rodbard D , et al. Comparison of insulin glargine 300 units/mL and 100 units/mL in adults with type 1 diabetes: continuous glucose monitoring profiles and variability using morning or evening injections. Diabetes Care. 2017;40(4):554‐560. 28115474 10.2337/dc16-0684

[52] Shama H . Statistical significance or clinical significance? A researcher's dilemma for appropriate interpretation of research results. Saudi J Anaesth. 2021;15(4):431‐434. 34658732 10.4103/sja.sja_158_21 PMC8477766

