# Supplementary material for: Use of basal insulin in the management of adults with type 2 diabetes: An Asia‐Pacific evidence‐based clinical practice guideline
Source: J Diabetes. 2023 Apr 23;15(6):474–87. doi: 10.1111/1753-0407.13392 (PMC10270740; doi:10.1111/1753-0407.13392)
Supplement: Supplementary file 1 — Appendix S1. Supplementary Information. [file JDB-15-474-s001.docx]

**Appendix Table S1. Vote results of the clinical threshold values for critical and important outcomes**

| **Outcomes** | **Trivial effect** | **Small effect** | **Moderate effect** | **Large effect** | **Agree** | **Disagree** | **Abstain** | **Agreement rate** |
| --- | --- | --- | --- | --- | --- | --- | --- | --- |
| **HbA_1c_ < 7%** | < 30 per 1000 | 30-50 per 1000 | >50-100 per 1000 | > 100 per 1000 | 15 | 0 | 1 | 94% |
| **Mean HbA_1c_ change value (from baseline to the end of the study), %** | < 0.4 | 0.4-1.0 | > 1.0-1.5 | > 1.5 | 15 | 1 | 0 | 94% |
| **Hypoglycemia < 3.0 mmol/L** | < 20 per 1000 | 20-50 per 1000 | >50-80 per 1000 | > 80 per 1000 | 15 | 0 | 1 | 94% |
| **Hypoglycemia < 3.9 mmol/L** | < 50 per 1000 | 50-100 per 1000 | >100-150 per 1000 | > 150 per 1000 | 15 | 0 | 1 | 94% |
| **Nocturnal hypoglycemia, hypoglycemia < 3.0 or 3.9 mmol/L** | < 20 per 1000 | 20-50 per 1000 | >50-80 per 1000 | > 80 per 1000 | 15 | 0 | 1 | 94% |
| **Severe hypoglycemia, hypoglycemia < 3.0 or 3.9 mmol/L** | < 5 per 1000 | 5-10 per 1000 | >10-20 per 1000 | > 20 per 1000 | 15 | 0 | 1 | 94% |
| **Mean FPG change value (from baseline to the end of the study)** | < 0.5 mmol/L | 0.5-1 mmol/L | >1-1.5 mmol/L | > 1.5 mmol/L | 14 | 1 | 1 | 88% |
| **Mean weight change (from baseline to the end of the study)** | < 2 % | 2 %-3 % | >3 %-5 % | > 5 % | 14 | 1 | 1 | 88% |

FPG, fasting plasma glucose.

**Appendix Table S2. The first online survey results by September 28, 2022**

| **Q1. What are the differences of the effectiveness and safety among 5 types of basal insulin regimens after the initiation of insulin therapy in adult patients with type 2 diabetes who require basal insulin therapy?** | | | | | | |
| --- | --- | --- | --- | --- | --- | --- |
| **Decision** | | Recommendation 1.1: Ultra-Long-acting insulin: between glargine U-300 and degludec U-100 at bedtime injection, we conditionally recommend glargine U-300. | Recommendation 1.2: Long-acting insulin: between glargine U-100 and detemir at bedtime injection, we conditionally recommended detemir. | Recommendation 1.3: Across Ultra-Long-acting and Long-acting insulins at bedtime injection, we conditionally recommend glargine U-300 or degludec U-100 when compared with glargine U-100. If morning injection is acceptable, glargine U-100 can be considered prioritized. | Recommendation 1.4: When glargine U-100 is the only option, we strongly recommend injecting it in the morning time rather than that at bedtime. | Recommendation 1.5: When Ultra-Long-acting insulin (glargine U-300 and degludec U-100) or long-acting insulin (glargine U-100 and detemir) are available, we strongly recommend choosing any of them compared with NPH insulin regardless morning time or bedtime injection. |
| **Agree** | | 8 | 8 | 8 | 5 | 12 |
| **Disagree** | | 4 | 4 | 5 | 8 | 2 |
| **Abstain** | | 2 | 2 | 1 | 1 | 0 |
| **Agreement rate** | | 57% | 57% | 57% | 36% | 86% |
| **Q2. What is the initial optimal dose (U/kg/day) for initiation of the 5 types of basal insulin regimens regarding morning or bedtime administration to control blood glucose with acceptable side effects for the target population?** | | | | | | |
| **Decision** | | Qualifying Statements 2.1: Recommended the initial dose for glargine U-300 was 0.20 U/kg/day. | Qualifying Statements 2.2: Recommended the initial dose for degludec U-100 was from 0.10 U/kg/day to 0.20 U/kg/day. | Recommendation 2.3: Recommended the initial dose for detemir was from 0.10 U/kg/day to 0.20 U/kg/day. Qualifying Statements: Base on research evidence and clinical experience, we choose "conditionally favour Detemir 0.12U/kg/day once daily at bedtime administration". | Recommendation 2.4: Recommended the initial dose for glargine U-100 was from 0.10 U/kg/day to 0.20 U/kg/day. Qualifying Statements: Based on research evidence and clinical experience, we choose "conditionally favour Glargine U-100 0.2 U/kg/day once daily at bedtime administration". | Qualifying Statements 2.5: Recommended the initial dose for NPH was from 0.11 U/kg/day to 0.23 U/kg/day. |
| **Agree** | | 8 | 13 | 11 | 11 | 5 |
| **Disagree** | | 5 | 1 | 3 | 3 | 5 |
| **Abstain** | | 1 | 0 | 0 | 0 | 4 |
| **Agreement rate** | | 57% | 93% | 79% | 79% | 36% |
| **Q3. In the target population who received any of the 5 types of insulin regimens, what is the optimal dose range (U/kg/day) of the basal insulin initiation that can lead to a satisfactory control of FPG?** | | | | | | |
| **Decision** | | Qualifying Statements 3.1: Recommended the endpoint dose for glargine U-300 was from 0.34 to 0.62 U/kg/day at bedtime administration. | Qualifying Statements 3.2: Recommended the endpoint dose for degludec U-100 was from 0.28 to 0.59 U/kg/day at bedtime administration. | Qualifying Statements 3.3: Recommended the endpoint dose for detemir was from 0.19 to 0.78 U/kg/day at bedtime administration. | Qualifying Statements 3.4: Recommended the endpoint dose for glargine U-100 was from 0.34 to 0.62 U/kg/day. | Qualifying Statements 3.5: Recommended the endpoint dose for NPH was from 0.19 to 0.66 U/kg/day at bedtime administration. |
| **Agree** | | 8 | 10 | 8 | 9 | 9 |
| **Disagree** | | 4 | 3 | 4 | 3 | 3 |
| **Abstain** | | 2 | 1 | 2 | 2 | 2 |
| **Agreement rate** | | 57% | 71% | 57% | 64% | 64% |
| **Q4. After initiation of any 5 types of basal insulin regimens, what range of controlled FPG can lead to the ideal HbA_1c_ level in the target population?** | | | | | | |
| **Decision** | Recommendation 4: Based on limited evidence and clinical experience, we conditionally recommend to reach the range of controlled FPG of 3.9-6.1 mmol/L for any basal insulin.to lead to the ideal HbA_1c_ level in adult patients with type 2 diabetes who require basal insulin therapy. However, for people who are at high risk of hypoglycemia (such as patients who have experienced hypoglycemia events or repeated hypoglycemia, or who are at high risk of hypoglycemia as judged by a doctor, such as the elderly over 65 years of age, or have comorbidities, frailty population), or people with low requirements for blood sugar control, such as severely ill patients, with short life expectancy, 3.9-7.0 mmol/L is recommended. | | | | | |
| **Agree** | 12 | | | | | |
| **Disagree** | 2 | | | | | |
| **Abstain** | 0 | | | | | |
| **Agreement rate** | 86% | | | | | |

FPG, fasting plasma glucose; NPH, Insulin Protamine Hagedorn; Q, question.

**Appendix Table S3. The second online survey results by October 21, 2022**

| **Q1. What are the differences in the effectiveness and safety among 5 types of basal insulin regimens after the initiation of insulin therapy in adult patients with type 2 diabetes who require basal insulin therapy?** | | | | | |
| --- | --- | --- | --- | --- | --- |
| **Decision** | Recommendation 1.1  (Conditional Recommendation): For ultra-long-acting insulin, between glargine U-300 and degludec U-100 at bedtime injection, glargine U-300 can be considered to be initiated first. Qualifying Statement: For patients with impaired kidney function or renal insufficiency, degludec U-100 may be considered to be initiated first. More high-quality clinical trials are required to investigate this issue. | Recommendation 1.2.  (Conditional Recommendation): For long-acting insulin, between glargine U-100 and detemir at bedtime injection, • For patients who have concern on hypoglycemia, detemir can be considered to be initiated first but a higher dose and often twice-a-day injection may be required. • For patients who prefer achieving HbA_1c_ < 7%, glargine U-100 can be considered to be initiated first. | Recommendation 1.3  (Conditional Recommendation): Across ultra-long-acting and long-acting insulin regimens at bedtime injection, glargine U-300 or degludec U-100 can be considered to be initiated first when compared with glargine U-100, according to acceptability and affordability. | Recommendation 1.4  (Conditional Recommendation): When ultra-long-acting insulin (glargine U-300 and degludec U-100) or long-acting insulin (glargine U-100 and detemir) regimens are available, we recommend choosing any of them rather than NPH insulin, according to acceptability and affordability. | Recommendation 1.5  (Conditional Recommendation): When glargine U-100 is the only option, morning injection instead of bedtime injection can be recommended according to patients’ acceptability. |
| **Agree** | 13 | 14 | 14 | 16 | 13 |
| **Disagree** | 1 | 2 | 1 | 0 | 0 |
| **Abstain** | 2 | 0 | 1 | 0 | 3 |
| **Agreement rate** | 81% | 88% | 88% | 100% | 81% |
| **Q2. What is the initial optimal dose (U/kg/day) for initiation of the 5 types of basal insulin regimens regarding morning or bedtime administration to control blood glucose with acceptable side effects for the target population?** | | | | | |
| **Decision** | Recommendation 2 (No Recommendation): There is insufficient evidence to clearly answer the Q2 to make recommendations. Qualifying Statements: The initial dose can be 0.10 to 0.20 U/kg/day for all five basal insulin regimens. For people with impaired kidney function or renal insufficiency, and the elderly over 65 years, dosage for any basal insulin regimen should be reduced as appropriate. | | | | |
| **Agree** | 16 | | | | |
| **Disagree** | 0 | | | | |
| **Abstain** | 0 | | | | |
| **Agreement rate** | 100% | | | | |
| **Q3. In the target population who received any of the 5 types of insulin regimens, what is the optimal dose range (U/kg/day) of the basal insulin initiation that can lead to a satisfactory control of FPG?** | | | | | |
| **Decision** | Recommendation 3 (No Recommendation): There is insufficient evidence to clearly answer the Q3 to make recommendations. Qualifying Statements: The maintenance dose is from 0.3 to 0.6 U/kg/day for all five basal insulin regimens. | | | | |
| **Agree** | 14 | | | | |
| **Disagree** | 1 | | | | |
| **Abstain** | 1 | | | | |
| **Agreement rate** | 88% | | | | |
| **Q4. After initiation of any 5 types of basal insulin regimens, what range of controlled FPG can lead to the ideal HbA_1c_ level in the target population?** | | | | | |
| **Decision** | Recommendation 4 (Conditional Recommendation): The range of controlled FPG of 3.9-6.1 mmol/L (70-110 mg/dL) can be recommended for any of five basal insulin regimens to achieve the ideal HbA_1c_ level in adult patients with type 2 diabetes who require basal insulin therapy. However, individuals with a high risk of hypoglycemia or individuals with low requirements for blood sugar control, such as severely ill patients, or individuals with short life expectancy, 3.9-7.0 mmol/L of FPG can be recommended. Individuals with a high risk of hypoglycemia may include patients who have experienced hypoglycemia events or repeated hypoglycemia, individuals at high risk of hypoglycemia as judged by a doctor, the elderly over 65 years, individuals with comorbidities, or the frailty population. | | | | |
| **Agree** | 14 | | | | |
| **Disagree** | 2 | | | | |
| **Abstain** | 0 | | | | |
| **Agreement rate** | 88% | | | | |

FPG, fasting plasma glucose; NPH, Insulin Protamine Hagedorn; Q, question.

**Appendix Table S4. One patient external reviewer’s review results**

| **Questions for Patients** | **Number (%)** | | | **Comments** |
| --- | --- | --- | --- | --- |
|  | **Yes** | **No** | **Unknown** |  |
| **1. Are the recommendations clear and unambiguous?** | 1 (100%) | 0 | 0 | None |
| **2. Do the recommendations reflect what the evidence shows?** | 1 (100%) | 0 | 0 | None |
| **3. Do the recommendations consider issues and/or address outcomes that are important to patients and members of the public?** | 1 (100%) | 0 | 0 | None |
| **4. Do the recommendations allow for flexibility based on patient preferences and individual needs?** | 1 (100%) | 0 | 0 | None |
| **5. Does the guideline recommend treatments or care practices that patients may consider unacceptable?** | 0 | 1 (100%) | 0 | None |
| **6. Do you have any comments on this guideline?** | NA | NA | NA | None |

NA, not applicable.

**Appendix Table S5. External review results from clinicians**

|  | **Number (%)** | | | | |
| --- | --- | --- | --- | --- | --- |
|  | Lowest Quality  (1) | (2) | (3) | (4) | Highest Quality  (5) |
| **1. Rate the overall quality of the guideline report** | 0 | 3 (9.10%) | 7 (21.2%) | 14 (42.4%) | 9 (27.3%) |
|  | Strongly Disagree  (1) | (2) | (3) | (4) | Strongly Agree  (5) |
| **2. I would make use of this guideline in my professional decisions.** | 0 | 4 (12.1%) | 9 (27.3%) | 11 (33.3%) | 9 (27.3%) |
| **3. I would recommend this guideline for use in practice.** | 0 | 4 (12.1%) | 6 (18.2%) | 14 (42.4%) | 9 (27.3%) |
| **4. What are the barriers or enablers to the implementation of this guideline in your clinical practice?** | 1) The cognitive level of primary endocrinologists on insulin therapy.  2) This guideline report has a certain guiding effect on the clinical application of basal insulin by non-Endocrinologists (such as GP, family doctors, et al). However, because most of the recommendations are conditional recommendations, they are of limited use to Endocrinologists.  3) Lack or limited availability of insulin analogues in government clinics and hospitals.  4) According to Thailand’s healthcare policy, the physician should start NPH as basal insulin first. If the patients had severe nocturnal hypoglycemia, the physician could switch to long/ultra-long-acting insulin later.  5) There are too many individual differences by race and age.  6) An individualized approach is ultimately necessary because many clinical factors (perceivable or unrecognized) influence the dosage and administration time of insulin therapy.  7) I think the socialisation of guidelines must be done every time/regularly to any doctor or medical person (nurses paramedics etc.) so they will know the latest guidelines.  8) There are some barriers such as irregular blood glucose detection, serious complications and poor self-care ability of the patients, which could affect the implementation of the guideline.  9) Complexity of diseases and the combination of oral hypoglycemic agents used.  10) Risk of hypoglycemia with a relatively low FPG target (70-110).  11) Barriers include the heterogeneity amongst the different countries within Asia-Pacific, particularly in healthcare reimbursement, medication cost, and availability of the different insulins.  12) The content of this guideline is more biased toward the overall concept rather than the details of clinical practice. | | | | |

**Appendix Table S6. Summary of the authors’ responses to main comments from external reviewers**

| **Comments** | **Responses** |
| --- | --- |
| 1. I can't get why U-100 was preferred to be used before breakfast rather than before bed. | Please have a look at Appendix Table S5. For glargine U-100, the evidence showed that morning injection may increase the number of patients who achieved HbA_1c_ < 7% and reduce nocturnal hypoglycemia events. However, the certainty of evidence is low and considering patients’ acceptability, we made a conditional recommendation. |
| 2. For ultra-long-acting basal insulin, an additional reason for preference to U-300 is required. I think both of them are similarly recommended. | If both glargine U-300 and degludec U-100 are available, a patient must choose one to start. We recommended that glargine U-300 can be considered to be initiated first because the evidence (Appendix Table S5) showed that glargine U-300 resulted in fewer hypoglycemia<3.0 mmol/L at 3 months, RR was 0.67 (95% CI 0.45 to 1.00), 39 fewer/1000 (64 fewer to 0 fewer); hypoglycemia<3.9 mmol/L at 3 months, RR was 0.87 (95% CI 0.77 to 0.99), 71 fewer/1000 (125 fewer to 5 fewer). Based on our clinical thresholds, both outcomes showed small effects and their 95% CIs crossed two clinical thresholds (small and moderate threshold lines), which meant that we may believe the effect results of these two outcomes. For nocturnal hypoglycemia<3.9 mmol/L at 3 months, it seems that evidence favored degludec U-100 with the RR of 1.09 (95% CI 0.95 to 1.25), 40 more patients/1000 (11 fewer to 100 more), which represented small effect. However, the 95% CI of the absolute value crossed three thresholds (small, moderate, and large effect threshold lines) and crossed the “0” line as well. Thus, the evidence is very uncertain about the effect of degludec U-100 on reducing nocturnal hypoglycemia<3.9 mmol/L at 3 months [52]. Since the certainty of the evidence is low, patients’ preferences should be respected. We have added this point to the recommendation. |
| 3. There is insufficient evidence for Recommendations 1.1, 1.2, 1.3, and 1.5. | Please see the response to Comment 2. above and the evidence in Appendix Table S5. Also, we have added several sentences to discuss the statistical significance and clinical significance under Discussion section. |
| 4. Recommendation 1.2 needs to be clarified, as to the statement of glargine being the choice for patients who prefer HbA_1c_ ＜7%, as A1C target is achieved with titration of insulin, not the type of insulin. | We have revised Recommendation 1.2 based on the reviewer’s comments. We also reorganized the sequence of the recommendations under Recommendation 1. |
| 5. Recommendation 1.2: Regarding hypoglycemia, 2nd generation basal insulin is safer than 1st generation basal insulin. I think there is no need to separate Recommendations 1.1 and 1.2. | Ultra-long-acting insulin (glargine U-300 and degludec U-100) and long-acting insulin (glargine U-100 and detemir) have different pharmacokinetic times and hypoglycemia risks. Thus, we prefer separating them. |
| 6. For Recommendation 1.2, I would suggest to remove “often” and add “to achieve an optimal HbA_1c._ | We have revised the original Recommendation 1.2 based on external reviewer’s comments. |
| 7. Why did you make conditional recommendation for Recommendation 1.3 “Across ultra-long-acting and long-acting insulin regimens at bedtime injection, glargine U-300 or degludec U-100 can be considered to be initiated first when compared with glargine U-100, according to acceptability and affordability.”? | Overall, the certainty of the evidence is low, and we considered that glargine U-300 or degludec U-100 may be unavailable in some places in some countries. However, after discussion, we have changed the wording of “can” to “should” to make this as a strong recommendation and added “availability” at the end. |
| 8. Whether 'time within the glucose target range, TIR' can also be used as a reference standard? | “Time in range” is one of our important outcomes at project plan. However, no eligible study reported it except for a trial with 50 patients to compare glargine U-300 with NPH [28]. Since so small sample size, the estimated effects had wide 95% CIs, which led to uncertainty of these results. |
| 9. Agree with most of the recommendation in research question 2-4, but do not agree with statements for research question1, and do not think this is the practice of most clinicians. | Clinical significance and statistical significance are different. “The statistically significant results may not of clinical importance, vice versa the results which are of clinical importance may not be statistically significant.” [51]. In the diabetes community, this is the first guideline to set up clinical thresholds regarding trivial, small, moderate, and large effects for every outcome based on the working group members’ clinical experience, and to make recommendations after balancing the magnitude of the beneficial and harmful effects of the outcomes based on these clinical thresholds. We completely understand some disagreement, and different researchers may set up slightly different clinical thresholds for the same outcomes. The most important and meaningfulness of our piece is that we keep our document transparent and clear, which will provide references for other investigators’ future research. |
| 10. I can't get why U-100 was preferred to be used before breakfast rather than before bed. | Please have a look at Appendix Table S5. For glargine U-100, the evidence showed that morning injection may increase the number of patients who achieved HbA_1c_ < 7% and reduce nocturnal hypoglycemia events. However, the certainty of the evidence is low, and considering patients’ acceptability, we made a conditional recommendation. |
| 11. Please can you consider rearranging the order of recommendations for research question 1 for better clarity? I suggest the following order: 1.5; 1.4; 1.3; 1.1; and 1.2. | After discussion, the working group members have reorganized recommendations 1.1 to 1.5, which can be easier for the intended guideline users to understand and follow these recommendations. |
| 12. For Recommendation 3 of request question 3, I think you can mention the overbasalization which includes using more than 0.5 IU/kg/day. Clinicians should be aware of overbasalization and that basal insulin has a 'celling effect'. | We have changed the range to 0.3 to 0.5U/kg/day based on several external reviewers’ comments, changed it from a qualifying statement to a conditional recommendation, and added two references to support this change. |
| 13. About the Research Question 3: Factors associated with the decision of the initial dose in addition to age and renal function (for example body weight, and baseline glycemic control condition, such as FBS and HbA_1c_) should also be taken into consideration. | We have added these considerations based on the reviewer’s comments. |
| 14. Reviewer 1. I think the target range for high-risk persons of hypoglycemia is too low in Recommendation 4. perhaps 3.9 mmol/L might be too low as a target for FBS, especially in high-risk groups. | In our Recommendation 4, we stated that “3.9-7.0 mmol/L of FPG can be considered” for high-risk persons of hypoglycemia. Thus, we did recommend reaching 3.9 mmol/L of FPG. Furthermore, we stated that “More high-quality clinical trials are required to investigate other targeted ranges of controlled FPG, such as 4.4-7.0 mmol/L.” Now, we have added “as clinically indicated” after “3.9-7.0 mmol/L of FPG can be considered”. Also, we have added the 2022 ADA statement regarding the expected range of time in range (3.9-10 mmol/L). |
| 15. I suggest the part about the individualized approach according to the patients' clinical circumstances should be emphasized a little more. | We have emphasized this point more and added “Patients’ value and preference need to be considered and respected.” in the recommendations. |
| 16. Please check if you included all the eligible papers in your systematic review. I found a current paper that included 12 RCTs for comparing detemir versus glargine (Rezaei S, et al. Expert Rev Clin Pharmacol 2022;15(6):767-77). | Our guideline focuses initiation of using basal insulins in T2DM, thus, most of the included papers in Rezaei 2022 did not meet our study selection criteria. However, the Meneghini 2013 paper was in our EndNote file, but was missed during our screening process. We have added it into our systematic review. Overall, we have 35 included papers. We have reviewed all the relevant places. |
| 17. "Practical Guidance on Basal Insulin Initiation and Titration in Asia: A Delphi-Based Consensus" is now available in Diabetes Therapy. Since there is no research question regarding basal insulin dose titration, it would give more complete information if this matter were added and cited in this new guideline. | We have cited this consensus guideline under the Introduction section. Mention. Some authors of that paper are our co-authors. |
| 18. (1) I would strongly recommend that this paper includes recognition of these challenges and variability amongst the different Asia-Pacific countries in the Limitations section or in the Introduction. 2) I note the limitation stated by the authors on not evaluating the cost-effectiveness of the five basal insulins. To consider whether guidelines are available in the respective countries of the key experts included in the Internal review and whether there is any need to mention the availability of these guidelines/ recommendations as national recommendations may have included country-specific cost-efficacy analysis in their recommendations. | We have added these points under the Limitation and Future Research sections. |
| 19. Notably, the guideline does not include several large countries in Asia-Pacific like India, the Philippines, etc., which may limit generalizability in these countries. | We agree with this comment and have added this point at the end of this document under GUIDELINE LIMITATIONS. |

**Appendix Table S7. Key evidence for Research Question 1: the effectiveness and safety of five basal insulins**

| Comparison | **N of studies with reference** | **Sample size** | **Favored insulin from a point estimate** | **Key evidence for the results with the point estimate of the critical outcome at least over the small effect thresholds** |
| --- | --- | --- | --- | --- |
| **1. One insulin bedtime injection vs. another insulin bedtime injection** | | | | |
| Glargine U-300 vs degludec U-100 | 1 (BRIGHT) (Rosenstock 2018, Cheng 2020) | 924 | Glargine U-300 | 1) HbA_1c_<7.0, % at 6 months, RR 1.09 (95% CI 0.95 to 1.25), 40 more patients per 1,000 reached HbA_1c_<7.0 % (22 fewer to 111 more).  2) Hypoglycemia<3.0 mmol/L at 3 months, RR 0.67 (95% CI 0.45 to 1.00), 39 fewer per 1,000 (64 fewer to 0 fewer).  3) Hypoglycemia<3.0 mmol/L at 6 months, RR 0.80 (95% CI 0.60 to 1.07), 37 fewer per 1,000 (74 fewer to 13 fewer).  4) Hypoglycemia<3.9 mmol/L at 3 months, RR 0.87 (95% CI 0.77 to 0.99), 71 fewer per 1,000 (125 fewer to 5 fewer). |
|  |  |  | Degludec U-100 | 1) Nocturnal hypoglycemia<3.9 mmol/L at 3 months, RR 1.24 (95% CI 0.93 to 1.66), 36 more per 1,000 (11 fewer to 100 more). |
|  | Subgroup analysis from BRIGHT (Bolli 2021) | 592 (Age < 65 years) | Glargine U-300 | 1. Hypoglycemia<3.0 mmol/Lat 3 months, RR 0.73 (95% CI 0.44 to 1.21), 30 fewer per 1,000 (62 fewer to 23 more). 2. Hypoglycemia<3.0 mmol/Lat 6 months, RR 0.73 (95% CI 0.51 to 1.05), 53 fewer per 1,000 (96 fewer to 10 more). 3. Hypoglycemia<3.9 mmol/Lat 3 months, RR 0.88 (95% CI 0.74 to 1.04), 61 fewer per 1,000 (131 fewer to 20 more). 4. Nocturnal hypoglycemia<3.9 mmol/Lat 3 months, RR 0.83 (95% CI 0.57 to 1.21), 30 fewer per 1,000 (75 fewer to 37 more). |
|  |  |  | Degludec U-100 | No critical outcome with a point estimate reached a small effect threshold. |
|  |  | 332 (Age ≥ 65 years) | Glargine U-300 | 1. Hypoglycemia<3.0 mmol/Lat 3 months, RR 0.56 (95% CI 0.29 to 1.11), 56 fewer per 1,000 (90 fewer to 14 more). 2. Hypoglycemia<3.9 mmol/Lat 3 months, RR 0.86 (95% CI 0.71 to 1.04), 86 fewer per 1,000 (178 fewer to 24 more). 3. Nocturnal hypoglycemia<3.9 mmol/L at 3 months, RR 0.76 (95% CI 0.48 to 1.20), 51 fewer per 1,000 (110 fewer to 42 more). |
|  |  |  | Degludec U-100 | 1. Nocturnal hypoglycemia<3.9 mmol/L at 6 months, RR 1.09 (95% CI 0.79 to 1.51), 26 more per 1,000 (61 fewer to 148 more). |
|  | Subgroup analysis (Haluzík 2020) | 467 (eGFR ≥90 mL/min/ 1.73m^2^) | Glargine U-300 | 1. Hypoglycemia<3.0 mmol/L at 6 months, RR 0.74 (95% CI 0.47 to 1.18), 41 fewer per 1,000 (84 fewer to 29 more). 2. Hypoglycemia<3.9 mmol/L at 6 months, RR 0.92 (95% CI 0.80 to 1.06), 52 fewer per 1,000 (130 fewer to 39 more). 3. Nocturnal Hypoglycemia<3.0 mmol/L at 6 months, RR 0.55 (95% CI 0.23 to 1.31), 26 fewer per 1,000 (45 fewer to 18 more). 4. Nocturnal Hypoglycemia<3.9 mmol/L at 6 months, RR 0.80 (95% CI 0.60 to 1.06), 64 fewer per 1,000 (129 fewer to 19 more). |
|  |  |  | Degludec U-100 | No outcome with a point estimate reached a small effect threshold. |
|  |  | 365 (eGFR 60-90 mL/ min/1.73m^2^) | Glargine U-300 | 1. Hypoglycemia<3.0 mmol/L at 6 months, RR 0.84 (95% CI 0.53 to 1.33), 30 fewer per 1,000 (88 fewer to 62 more). |
|  |  |  | Degludec U-100 | 1. Nocturnal Hypoglycemia<3.0 mmol/L at 6 months, RR 1.68 (95% CI 0.70 to 4.02), 28 more per 1,000 (12 fewer to 125 more). 2. Nocturnal Hypoglycemia<3.9 mmol/L at 6 months, RR 1.27 (95% CI 0.90 to 1.78), 64 more per 1,000 (24 fewer to 186 more). |
|  |  | 96 (eGFR <60 mL/min/ 1.73m^2^) | Glargine U-300 | 1. HbA_1c_ change at 6 months was 0.43% lower (95% CI -0.74 to -0.12%). 2. Event rate/patient-year Nocturnal Hypoglycemia <3.0 mmol/L at 6 months, RR 0.80 (95% CI 0.37 to 1.74) (no sufficient data to calculate AE). 3. Event rate/patient-year Nocturnal Hypoglycemia <3.9 mmol/L at 6 months, RR 0.68 (95% CI 0.22 to 2.09), (no sufficient data to calculate AE). |
|  |  |  | Degludec U-100 | 1. Nocturnal Hypoglycemia<3.0 mmol/L at 6 months, RR 1.19 (95% CI 0.47 to 3.03), 27 more per 1,000 (76 fewer to 290 more). 2. Nocturnal Hypoglycemia<3.9 mmol/L at 6 months, RR 1.17 (95% CI 0.68 to 2.02), 56 more per 1,000 (104 fewer to 333 more). |
| Glargine U-100 vs Detemir | 4 (Rosenstock 2008, Elisha 2015, Cander 2014, Meneghini 2013) | 1119 | Glargine U-100 | 1) HbA_1c_<7.0% at 6 months, RR 1.37 (95% CI 1.10 to 1.70), 142 more per 1,000 (38 more to 268 more).  2) HbA_1c_ change, % at 12 months, MD -0.42 (95% CI -1.11 to 0.27).  3) Nocturnal Hypoglycemia<3.0 mmol/L at 6 months, RR 0.65 (95% CI 0.38 to 1.13), 45 fewer per 1,000 (80 fewer to 17 more). |
|  |  |  | Detemir | 1) Hypoglycemia<3.0 mmol/L at 6 months, RR 1.33 (95% CI 0.98 to 1.79), 79 more per 1,000 (5 fewer to 189 more).  2) Hypoglycemia<3.0 mmol/L at 12 months, RR 1.12 (95% CI 0.95 to 1.32), 56 more per 1,000 (23 fewer to 148 more). |
| Degludec U-100 vs Glargine U-100 | 3 (Onishi 2013, Pan 2016, Zinman 2012) | 2298 | Degludec U-100 | 1) Severe hypoglycemia at 12 months, RR 0.13 (95% CI 0.03 to 0.69), 17 fewer per 1,000 (19 fewer to 6 fewer).  2) Nocturnal hypoglycemia<3.0 mmol/L at 6 months, RR 0.83 (95% CI 0.62 to 1.12), 24 fewer per 1,000 (54 fewer to 17 more).  3) Hypoglycemia<3.0 mmol/L at 6 months, RR 0.89 (95% CI 0.76 to 1.03), 41 fewer per 1,000 (89 fewer to 11 more). |
|  |  |  | Glargine U-100 | No critical outcome with a point estimate reached a small effect threshold. |
| Glargine U-300 vs Glargine U-100 | **3** (Bolli 2015, Bolli 2017, Ji 2020) | 1482 | Glargine U-300 | 1) HbA_1c_<7.0 % at 12 months, RR 1.23 (95% CI 0.98 to 1.54), 54 more per 1,000 (5 fewer to 127 more).  2) Hypoglycemia<3.0 mmol/L at 12 months, RR 0.63 (95% CI 0.47 to 0.84), 82 fewer per 1,000 (117 fewer to 35 fewer).  3) Hypoglycemia<3.0 mmol/L at 3 months, RR 0.83 (95% CI 0.73 to 0.94), 118 fewer per 1,000 (187 fewer to 41 fewer).  4) Nocturnal hypoglycemia<3.9 mmol/Lat 3 months, RR 0.67 (95% CI 0.51 to 0.88), 110 fewer per 1,000 (163 fewer to 40 fewer).  5) Hypoglycemia<3.9 mmol/Lat 6 months, RR 0.91 (0.84 to 0.99), 53 fewer per 1,000 (94 fewer to 6 fewer).  6) Nocturnal hypoglycemia<3.9 mmol/Lat 6 months, RR 0.81 (95% CI 0.70 to 0.94), 61 fewer per 1,000 (96 fewer to 19 fewer).  7) Nocturnal hypoglycemia<3.9 mmol/Lat 12 months, RR 0.86 (95% CI 0.69 to 1.07), 41 fewer per 1,000 (90 fewer to 20 more). |
|  |  |  | Glargine U-100 | No critical outcome with a point estimate reached a small effect threshold. |
| Glargine U-300 vs NPH | 1 (Ling 2021) | 50 | Glargine U-300 | 1) Hypoglycemia<3.0 mmol/L at 6 months, RR 0.20 (95% CI 0.03 to 1.58), 174 fewer per 1,000 (211 fewer to 126 more).  2) Hypoglycemia<3.9 mmol/L at 3 months, RR 0.41 (95% CI 0.21 to 0.80), 436 fewer per 1,000 (584 fewer to 148 fewer).  3) Hypoglycemia<3.9 mmol/L at 6 months, RR 0.35 (95% CI 0.17 to 0.73), 480 fewer per 1,000 (613 fewer to 200 fewer).  4) Nocturnal hypoglycemia<3.0 mmol/L at 6 months, RR 0.11 (95% CI 0.01 to 1.95), 155 fewer per 1,000 (172 fewer to 165 more).  5) Nocturnal hypoglycemia<3.9 mmol/L at 3 months, RR 0.05 (95% CI 0.00 to 0.77), 413 fewer per 1,000 (∞ to 100 fewer).  6) Nocturnal hypoglycemia<3.9 mmol/L at 6 months, RR 0.11 (95% CI 0.02 to 0.81), 348 fewer per 1,000 (383 fewer to 74 fewer). |
|  |  |  | NPH | No critical outcome with a point estimate reached a small effect threshold. |
| Glargine U-100 vs NPH | 12 (Hermanns 2015, Yki-Jarvinen 2006, Pan 2007, Forst 2010, Riddle 2003, Fritsche 2003, Eliaschewitz 2006, Oikonomou 2014, Benedetti 2003, Yki-Jarvinen 2000, Hsia 2011, Home 2015) | 4233 | Glargine U-100 | 1) HbA_1c_<7.0 % at 6 months, RR 1.07 (95% CI 0.99 to 1.14), 31 more per 1,000 (4 fewer to 63 more).  2) Hypoglycemia<3.0 mmol/L at 3 months, RR 0.87 (95% CI 0.37 to 2.02), 52 fewer per 1,000 (250 fewer to 405 more).  3) Nocturnal hypoglycemia<3.9 mmol/L at 6 months, RR 0.58 (95% CI 0.47 to 0.73), 143 fewer per 1,000 (180 fewer to 92 fewer).  4) Nocturnal hypoglycemia<3.0 mmol/L at 12 months, RR 0.42 (95% CI 0.30 to 0.58), 139 fewer per 1,000 (168 fewer to 101 fewer). |
|  |  |  | NPH | 1) Hypoglycemia<3.0 mmol/L at 6 months, RR 1.21 (95% CI 0.75 to 1.95), 32 more per 1,000 (38 fewer to 144 more). |
| Detemir vs NPH | 1 (Tsimikas 2006) | 333 | Detemir | 1) Hypoglycemia<3.0 mmol/L at 6 months, RR 0.49 (95% CI 0.33 to 0.75), 165 fewer per 1,000 (217 fewer to 81 fewer).  2) Nocturnal hypoglycemia<3.0 mmol/L at 6 months, RR 0.35 (95% CI 0.16 to 0.77), 87 fewer per 1,000 (113 fewer to 31 fewer). |
|  |  |  | NPH | No critical outcome with a point estimate reached a small effect threshold. |
| **2**. **One insulin morning vs another insulin morning injection** | | | | |
| **Degludec U-100 vs Glargine U-100** | **1 (**Aso 2017) | 44 | Degludec U-100 | No critical outcome with a point estimate reached a small effect threshold. |
|  |  |  | Glargine U-100 | 1. HbA_1c_ Endpoint, % at 6 months, MD 0.2 (95% CI -0.35 to 0.75). |
| Detemir vs NPH | 1 (NCT00506662) | 86 | Detemir | 1) Total hypoglycaemic episodes (rate/week) at 7 months (mean ± SD):  Detemir vs NPH: 0.079 ± 0.359 vs 0.146 ± 0.743.  2) Minor hypoglycaemic episodes (rate/week): Detemir vs NPH: 0 vs 0.125 ± 0.733. |
|  |  |  | NPH | No critical outcome with a point estimate reached a small effect threshold. |
| **3. One insulin morning injection vs another insulin bedtime injection** | | | | |
| Detemir Morning time vs NPH Bedtime | 1 (Tsimikas 2006) | 329 | Detemir | 1. Hypoglycemia<3.0 mmol/L at 6 months, RR 0.60 (95% CI 0.41 to 0.88), 129 fewer per 1,000 (191 fewer to 39 fewer). 2. Nocturnal hypoglycemia<3.0 mmol/L at 6 months, RR 0.18 (95% CI 0.06 to 0.51), 110 fewer per 1,000 (126 fewer to 66 fewer). |
|  |  |  | NPH | No critical outcome with a point estimate reached a small effect threshold. |
| Glargine U-100 Morning time vs NPH Bedtime | 2 (Fritsche 2003, Hsia 2011) | 526 | Glargine U-100 | 1. HbA_1c_<7.0 % at 6 months, RR 1.34 (95% CI 1.07 to 1.69), 105 more per 1,000 (22 more to 213 more). 2. Nocturnal hypoglycemia<3.9 mmol/L at 6 months, RR 0.43 (95% CI 0.31 to 0.60), 219 fewer per 1,000 (265 fewer to 153 fewer). |
|  |  |  | NPH | No critical outcome with a point estimate reached a small effect threshold. |
| **4. Same insulin morning time injection vs bedtime injection** | | | | |
| **Detemir** | 1 (Tsimikas 2006) | 334 | Morning time | 1. Nocturnal hypoglycemia<3.0 mmol/L at 6 months, RR 0.51 (95% CI 0.16 to 1.67), 23 fewer per 1,000(40 fewer to 32 more). 2. Severe hypoglycemia at 6 months, RR 0.20 (95% CI 0.01 to 4.23), 9 more per 1,000 (12 fewer to 38 more). |
|  |  |  | Bedtime | 1. Hypoglycemia<3.0 mmol/L at 6 months, RR 1.21 (95% CI 0.76 to 1.93), 34 more per 1,000 (38 fewer to 149 more). |
| **Glargine U-100** | 2 (Fritsche 2003, Hsia 2011) | 521 | Morning time | 1) HbA_1c_<7.0 % at 6 months, RR 1.30 (95% CI 1.04 to 1.63), 96 more per 1,000 (13 more to 201 more).  2) HbA_1c_ Change, % at 6 months, MD -0.54 (95% CI -1.16 to 0.09).  3) Nocturnal hypoglycemia<3.9 mmol/L at 6 months, RR 0.72 (95% CI 0.50 to 1.05), 64 fewer per 1,000 (115 fewer to 11 more). |
|  |  |  | Bedtime | No critical outcome with a point estimate reached a small effect threshold. |

AE, absolute effect; eGRF, estimated glomerular filtration rate; MD, mean difference; NPH, Insulin Protamine Hagedorn; RR, risk ratio; SD, standard deviation; CI, confidence interval.

**Appendix Table S8. Key evidence for Research Question 4: the range of the target FPG can lead to the ideal HbA_1c_ level (i.e., < 7·0%)**

| **N of studies with reference** | **Comparison** | **Sample size** | **Favored insulin from point estimate** | **K**ey e**vidence for the results with the point estimate of the critical outcome over the small effect thresholds** |
| --- | --- | --- | --- | --- |
| **Determir** | | | | |
| 1 (Blonde 2009) | 3.9 < FPG ≤ 5.0 mmol/L vs. 4.4< FPG ≤ 6.1 mmol/L | 244 | 3.9 < FPG ≤ 5.0 mmol/L | 1) HbA_1c_ < 7% at 6 months, RR 1.19 (95% CI 0.97 to 1.47), 103 more per 1,000 (16 fewer to 254 more). |
|  |  |  | 4.4< FPG ≤ 6.1 mmol/L | 1) Hypoglycemia (not report critical value) at 6 months, RR 1.27 (95% CI 0.97 to 1.67), 111 more per 1,000 (12 fewer to 275 more).  2) Nocturnal Hypoglycemia (not report critical value) at 6 months, RR 1.49 (95% CI 0.96 to 2.32), 100 more per 1,000 (8 fewer to 270 more). |
| **Glargine U-100** | | | | |
| 2 (Yuan 2021; Yang 2019） | 3.9 < FPG ≤ 5.6 mmol/L vs. 3.9 < FPG ≤ 6.1 mmol/L | 577 | 3.9 < FPG ≤ 5.6 mmol/L | No critical outcome with a point estimate reached a small effect threshold. |
|  |  |  | 3.9 < FPG ≤ 6.1 mmol/L | 1) HbA_1c_ < 7% at 6 months, RR 0.87 (95% CI 0.46 to 1.65), 59 fewer per 1,000 (244 fewer to 293 more).  2) Hypoglycemia ≤3.9 mmol/L at 6 months, RR 1.42 (95% CI 1.08 to 1.86), 115 more per 1,000 (22 more to 236 more).  3) Nocturnal Hypoglycemia ≤3.9 mmol/L at 6 months, RR 1.86 (95% CI 1.18 to 2.92), 92 more per 1,000 (19 more to 205 more). |
| 2 (Yuan 2021; Yang 2019） | 3.9 < FPG ≤ 6.1 mmol/L vs. 3.9 < FPG ≤ 7.0 mmol/L | 872 | 3.9 < FPG ≤ 6.1 mmol/L | 1) HbA_1c_ < 7% at 6 months, RR 1.24 (95% CI 1.05 to 1.45), 87 more per 1,000 (18 more to 162 more). |
|  |  |  | 3.9 < FPG ≤ 7.0 mmol/L | 1) Nocturnal Hypoglycemia ≤ 3.9 mmol/L at 6 months, RR 1.24 (95% CI 0.81 to 1.91), 21 more per 1,000 (16 fewer to 78 more). |
| 2 (Yuan 2021; Yang 2019） | 3.9 < FPG ≤ 5.6 mmol/L vs. 3.9 < FPG ≤ 7.0 mmol/L | 587 | 3.9 < FPG ≤ 5.6 mmol/L | 1) HbA_1c_ < 7% at 6 months, RR 1.17 (95% CI 0.93 to 1.47), 61 more per 1,000 (25 fewer to 169 more). |
|  |  |  | 3.9 < FPG ≤ 7.0 mmol/L | 1) Nocturnal Hypoglycemia ≤3.9 mmol/L at 6 months, RR 2.31 (95% CI 1.43 to 3.71), 113 more per 1,000 (37 more to 233 more).  2) Hypoglycemia ≤3.9 mmol/L at 6 months, RR 1.67 (95% CI 1.26 to 2.22), 156 more per 1,000 (61 more to 284 more). |

FPG, fasting plasma glucose; RR, risk ratio; CI, confidence interval.
